# The NKCC1 ion transporter modulates microglial phenotype and inflammatory response to brain injury in a cell-autonomous manner

**DOI:** 10.1371/journal.pbio.3001526

**Published:** 2022-01-27

**Authors:** Krisztina Tóth, Nikolett Lénárt, Péter Berki, Rebeka Fekete, Eszter Szabadits, Balázs Pósfai, Csaba Cserép, Ahmad Alatshan, Szilvia Benkő, Dániel Kiss, Christian A. Hübner, Attila Gulyás, Kai Kaila, Zsuzsanna Környei, Ádám Dénes

**Affiliations:** 1 Momentum Laboratory of Neuroimmunology, Institute of Experimental Medicine, Budapest, Hungary; 2 János Szentágothai Doctoral School of Neurosciences, Semmelweis University, Budapest, Hungary; 3 Laboratory of Cerebral Cortex Research, Institute of Experimental Medicine, Budapest, Hungary; 4 Department of Physiology, Faculty of Medicine, University of Debrecen, Debrecen, Hungary; 5 Doctoral School of Molecular Cellular and Immune Biology, Faculty of Medicine, University of Debrecen, Debrecen, Hungary; 6 Software Engineering Institute, John von Neumann Faculty of Informatics, Óbuda University, Budapest, Hungary; 7 University Hospital Jena, Friedrich Schiller University, Jena, Germany; 8 Molecular and Integrative Biosciences and Neuroscience Center (HiLIFE), University of Helsinki, Helsinki, Finland; Charite Universitatsmedizin Berlin, GERMANY

## Abstract

The NKCC1 ion transporter contributes to the pathophysiology of common neurological disorders, but its function in microglia, the main inflammatory cells of the brain, has remained unclear to date. Therefore, we generated a novel transgenic mouse line in which microglial NKCC1 was deleted. We show that microglial NKCC1 shapes both baseline and reactive microglia morphology, process recruitment to the site of injury, and adaptation to changes in cellular volume in a cell-autonomous manner via regulating membrane conductance. In addition, microglial NKCC1 deficiency results in NLRP3 inflammasome priming and increased production of interleukin-1β (IL-1β), rendering microglia prone to exaggerated inflammatory responses. In line with this, central (intracortical) administration of the NKCC1 blocker, bumetanide, potentiated intracortical lipopolysaccharide (LPS)-induced cytokine levels. In contrast, systemic bumetanide application decreased inflammation in the brain. Microglial NKCC1 KO animals exposed to experimental stroke showed significantly increased brain injury, inflammation, cerebral edema and worse neurological outcome. Thus, NKCC1 emerges as an important player in controlling microglial ion homeostasis and inflammatory responses through which microglia modulate brain injury. The contribution of microglia to central NKCC1 actions is likely to be relevant for common neurological disorders.

## Abstract 2

This study shows that inflammatory responses of microglial cells are markedly influenced by the ion transporter, NKCC1; blockade or genetic deletion of microglial NKCC1 has broad cell-autonomous effects, leading to changes in morphology, membrane conductance, process recruitment after injury, and cytokine production, with worsened neurological outcome after stroke.

## Introduction

Members of the plasmalemmal cation-chloride cotransporter (CCC) family, such as the neuron-specific K^+^/Cl^−^ extruder, KCC2, and the ubiquitously expressed Na^+^-K^+^-2Cl^−^ cotransporter, NKCC1 (coded by the *Slc12a2* gene), have received a steeply increasing amount of attention in research on central nervous system (CNS) diseases, ranging from neuropsychiatric diseases to epilepsy, stroke, and dementia [1–6]. Notably, there is an abundance of studies, which have shown that the mRNA and protein expression levels as well as the functionality of NKCC1 are enhanced in injured and posttraumatic neurons [7,8]. This, in turn, has raised the obvious possibility that assuming a pathophysiological role for NKCC1, therapeutic actions might be achieved by pharmacological inhibition of this transporter. Indeed, data in a number of experimental studies based on systemic application of the selective NKCC1 blocker, the loop diuretic bumetanide, have suggested that this drug has therapeutic actions in diverse neuropathological conditions. These include neonatal seizures, temporal lobe epilepsy, autism spectrum disorders, schizophrenia, and brain edema after traumatic or hypoxic/ischemic injury [1–4,7–12]. In most of these studies, bumetanide has been suggested to exert its therapeutic effects by acting directly on NKCC1 expressed in central neurons.

While bumetanide is routinely used to block neuronal NKCC1 in experiments performed in vitro [3,8,12], the view that the drug might have direct effects on central neurons in vivo has raised numerous questions. First, the poor pharmacokinetic properties of bumetanide, including a low penetration across the blood–brain barrier (BBB) implies that when applied systemically at clinically relevant doses, the drug may not reach pharmacologically relevant concentrations in the brain parenchyma, a prediction that has been experimentally verified [10,13,14]. Another important point in the present context is that NKCC1 is expressed within the immature and mature CNS mainly in nonneuronal cell types [15], such as oligodendrocytes and their precursors [16,17], ependymal cells, and astrocytes [18–21]. Thus, even if applied directly into brain tissue, bumetanide or any other NKCC1 blockers would be expected to inhibit this ion transporter in a wide variety of cell types.

Interestingly, common neurological disorders including those with altered NKCC1 activity [6,7,22] display broad neuroinflammatory changes. While the impact of NKCC1 function on inflammatory cytokine production has not been widely studied, emerging evidence indicates up-regulated NKCC1 expression in response to inflammatory stimuli (LPS, IL-1β, TNF-α) [23–26]. Notably, while microglia are the main inflammatory cell type in the CNS, there is no information about the functional role of NKCC1 in microglia and whether microglial NKCC1 could provide a significant contribution to central NKCC1 actions under inflammatory conditions or after brain injury.

In the healthy brain, microglia regulate a wide variety of neuronal functions, whereas altered microglial activity is linked with the pathophysiology of most common brain disorders, such as neurodegenerative and psychiatric diseases, stroke, and epilepsy [27–33]. Extracellular accumulation of potassium is related to diverse neuropathological alterations [32,34], and the contribution of microglial potassium channels and transporters to microglial activity is widely recognized. These include membrane-expressed potassium channels (Kv1.3, THIK-1, Kir2.1) that regulate microglial motility, immune surveillance, and cytokine release, among others [35,36]. Moreover, changes in intracellular potassium and chloride levels are associated with inflammasome activation contributing to the regulation of interleukin-1β (IL-1β) release [37–39]. Interestingly, recent transcriptomic data verified high-level NKCC1 expression in microglia [21,40]. However, currently no experimental data are available on the function of microglial NKCC1 under physiological and pathological conditions. To this end, we studied whether central and systemic NKCC1 blockade impact on central inflammatory changes differently and tested the hypothesis that NKCC1 is involved in the regulation of microglial inflammatory mediator production, neuroinflammation, and brain injury.

## Results

### Systemic and central blockade of NKCC1 regulate LPS-induced inflammatory cytokine production in the brain in an opposite manner

**Fig 1:**
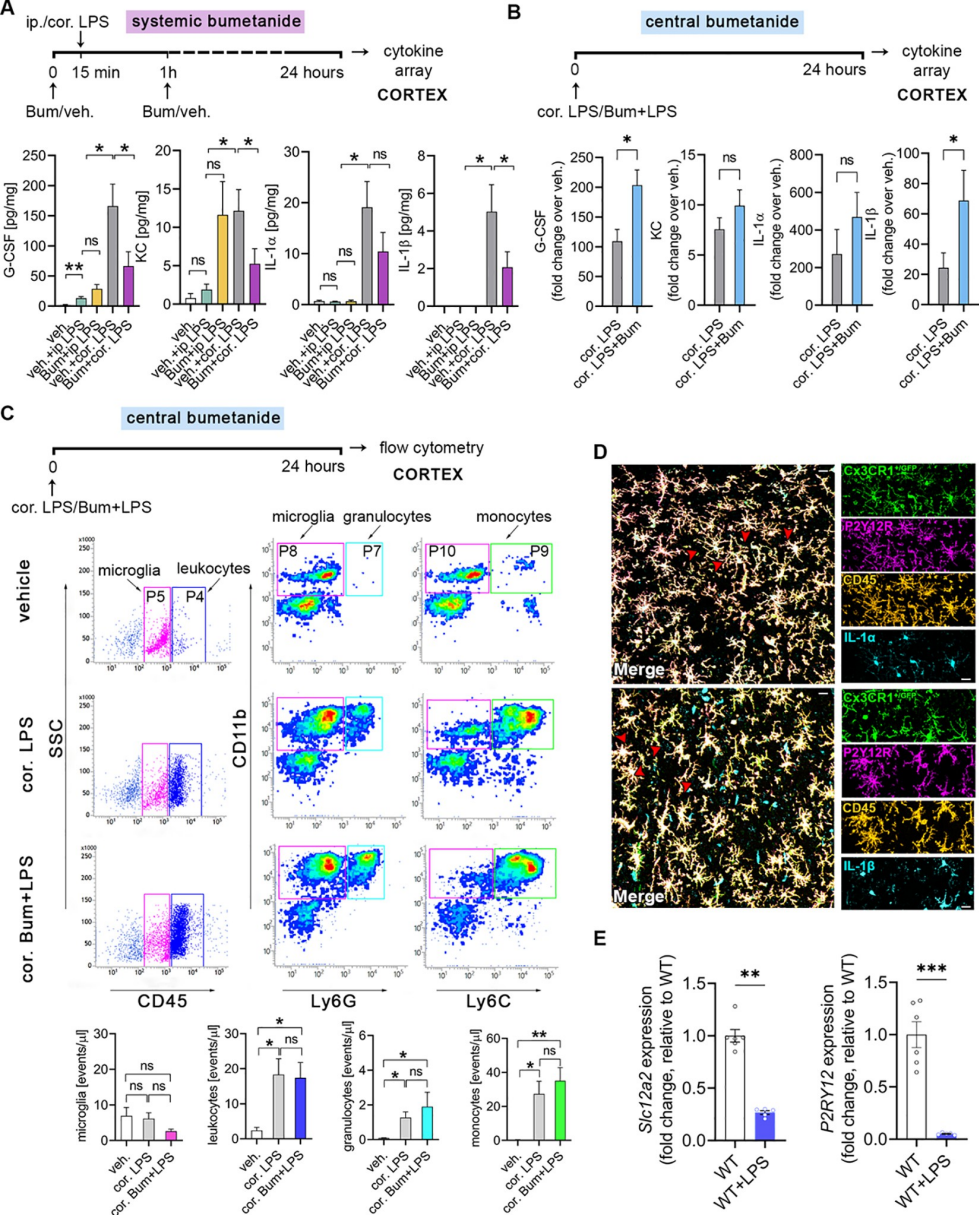
Systemic and central (intracortical) blockade of NKCC1 regulate LPS-induced inflammatory cytokine production in the brain in an opposite manner. **(A)** Mice were subjected to either ip. or cor. LPS injections, while NKCC1 was blocked by ip. Bum administration. Central LPS injection triggers high cytokine (G-CSF, IL-1α, IL-1β) and KC responses in the brain compared to ip. LPS injection, which is blocked by ip. Bum administration. (**B)** Central NKCC1 inhibition by cor. Bum administration significantly increases G-CSF and IL-1β levels. See also S1 Fig for effects of systemic vs. central blockade of NKCC1 on LPS-induced cytokine responses in the periphery. (**C)** Flow cytometric dot plots show that cortical administration of Bum does not affect the number of microglia (CD45^int^/P5 gate), and recruitment of leukocytes (CD45^high^/P4 gate), including monocytes (CD11b^+^, Ly6C^high^ /P9 gate), and granulocytes (CD11b^+^, Ly6G^high^/P7 gate) upon central LPS injection. (**D)** The main source of IL-1α and IL-1β in the brain are microglia cells. Confocal images of Cx3CR1^+/GFP^ brain slices show IL-1α-CD45-P2Y12R (above, red arrowheads) and IL-1β-CD45-P2Y12R (below, red arrowheads) labeled cells after cortical LPS injection-induced inflammation. (**E)** NKCC1 (encoded by *Slc12a2*) and P2Y12R gene expression is down-regulated in microglia isolated from adult mice 24 hours after cisterna magna LPS application. (**A)** Kruskall–Wallis followed by Dunn’s multiple comparison test; *p < 0.05; *N* (veh.) = 5, *N* (veh. + ip. LPS) = 5, *N* (Bum + ip. LPS) = 5, *N* (veh. + cor. LPS) = 5, *N* (Bum + cor. LPS) = 9. (**B)** Unpaired *t* test; *p < 0.05; *N =* 9/group; data were pooled from two independent studies. (**C)** One-way ANOVA followed by Tukey’s multiple comparison test; *p < 0.05; *N* (veh.) = 5, *N* (cor. LPS) = 6, *N* (cor. Bum + LPS) = 6. (**D)** Scale: 25 μm. (**E)** Unpaired *t* test; **p < 0.01, ***p < 0.001; *N* (WT) = 6, *N* (WT + LPS) = 5. Data underlying this figure can be found in S1 Data. Bum, bumetanide; cor., cortical; ip., intraperitoneal; ns, not significant; veh., vehicle; WT, wild type.

To investigate whether systemic blockade of NKCC1 actions by bumetanide could alter inflammatory responses in the CNS, we injected mice either intraperitoneally or intracortically with bacterial lipopolysaccharide (LPS), while NKCC1 actions were blocked by systemic (intraperitoneal (ip.), 2 mg/kg) bumetanide administration (Fig 1A). As expected, LPS administration by ip. injection triggered marked inflammation in the spleen and the liver (S1A Fig), but caused low cytokine production in cortical brain tissues, which was not influenced by ip. bumetanide treatment (Fig 1A). However, bumetanide resulted in a small but significant increase in LPS-induced IL-1β production in the spleen (S1A Fig). In contrast, intracortical LPS administration triggered a robust inflammatory response in the brain as seen earlier [41], which was reduced by ip. bumetanide treatment. This was demonstrated by lower G-CSF, KC, IL-1β, and IL-1α levels in the brain (by 39.8%, 43%, 54.6%, and 41%, respectively), while systemic cytokine levels were not altered (S1A Fig).

To compare the effects of systemic versus central NKCC1 blockade by bumetanide, we next investigated the impact of central (intracortical) bumetanide administration on LPS-induced cytokine responses, by coinjecting bumetanide with LPS (50 μM bumetanide in 200 nl final volume, rate: 200 nl/10 minutes; Fig 1B) into the cerebral cortex. As expected, intracortical LPS without bumetanide resulted in a 20- to 50-fold increase in inflammatory cytokines/chemokines in the cerebral cortex compared to vehicle. Surprisingly, on top of this, bumetanide markedly potentiated LPS-induced G-CSF, KC, IL-1β, and IL-1α levels in the brain (by 86.1%, 31.2%, 82.5%, and 72.4%, respectively), in sharp contrast with the effects of ip. bumetanide treatment (Fig 1A). Intracortical bumetanide had no effect on systemic cytokine levels (S1B Fig).

We next tested whether the effects of central bumetanide administration on increasing cytokine production might be explained by altered LPS-induced recruitment of leukocytes. However, while flow cytometry revealed increased number of infiltrating CD45^high^ leukocytes, in particular CD11b^+^ Ly6G^high^ granulocytes, and CD11b^+^ Ly6G^−^ Ly6C^high^ monocytes in response to central LPS injection compared to vehicle administration (Fig 1C), this was not altered by central bumetanide. The number of CD45^int^ CD11b^+^ microglia was not affected either (Fig 1C), indicating that the increased LPS-induced cytokine production observed upon central bumetanide treatment is not due to altered inflammatory cell numbers in the cerebral cortex. Unbiased densitometric analysis revealed that GFAP and AQP4 immunopositivity 24 hours after intracortical LPS injection were not altered by central bumetanide treatment (S2 Fig), suggesting that while bumetanide may also act on astroglial NKCC1 [18–20], marked changes in astrocyte phenotypes or perivascular astrocyte endfeet are unlikely to explain the effect of bumetanide on central inflammatory responses in the present study.

Microglia are the primary source of inflammatory cytokines in a number of neuropathologies, and IL-1β, also produced by microglia, is a key proinflammatory cytokine regulating the brain’s cytokine network [42,43]. To investigate the cellular source of IL-1β in this experimental model, we injected LPS intracortically into Cx3CR1^+/GFP^ (microglia reporter) mice. Multilabel immunostainings confirmed that Cx3CR1-GFP microglia coexpressing P2Y12R and CD45 displayed cytoplasmic IL-1α and IL-1β production (Fig 1D). These findings collectively suggested that microglia are likely to be major mediators of central bumetanide actions under inflammatory conditions. Supporting this, central LPS administration resulted in a significant decrease of NKCC1 (encoded by *Slc12a2*) mRNA levels, along with a decline of P2Y12R mRNA, a key purinergic receptor reportedly associated with homeostatic microglial actions, microglial activation, and response to injury (Fig 1E) [31,44–46].

### Deletion of microglial NKCC1 markedly impacts on baseline cell morphology and alters transformation to reactive microglia

**Fig 2:**
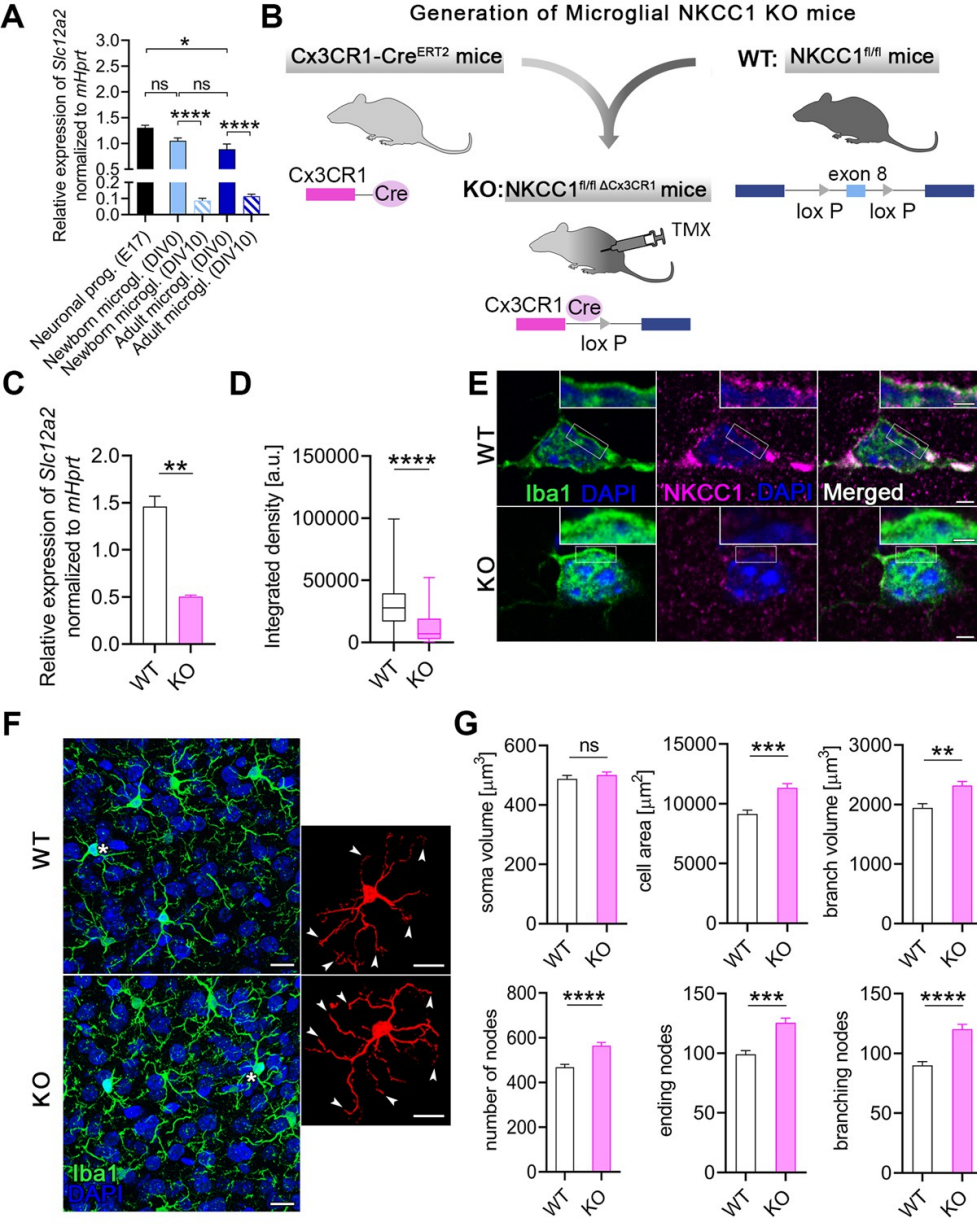
Deletion of microglial NKCC1 markedly impacts on baseline cell morphology and alters transformation to reactive microglia. **(A)** NKCC1 mRNA expression levels in newborn and adult microglia isolated from C57BL/6J mice compared to neural progenitors derived from E17 embryonic hippocampi. Note that NKCC1 mRNA levels decrease dramatically during in vitro maintenance (DIV10). (**B)** We generated a novel microglia-specific conditional NKCC1 KO transgenic mouse line by crossing NKCC1^fl/fl^ (exon 8 of the *Slc12a2* gene was flanked with lox P sites) and Cx3CR1-Cre^ERT2^ mice. (**C)** NKCC1 mRNA levels in isolated NKCC1 KO microglia was markedly reduced in comparison to WT cells. (**D, E)** NKCC1 protein expression in a large number of randomly sampled NKCC1 KO microglia cells is markedly reduced compared to WT cells. Inserts show plasma membrane localization of NKCC1. (**F, G)** Automated morphological analysis and maximum intensity projections of confocal images. Inserts show cells marked with white asterisks in 3D. Arrowheads indicate altered branch structure of NKCC1 KO microglia. Automated morphological analysis shows that features of NKCC1-deficient microglia significantly differ from WT microglia. **(A)** One-way ANOVA, followed by Holm–Sidak’s post hoc test. *N* (Neuronal progenitor) = 3, *N* (Newborn microglia DIV0) = 6, *N* (Newborn microglia DIV10) = 5, *N* (Adult microglia DIV0) = 7, *N* (Adult microglia DIV10) = 6. **: *p* < 0.01. (**C)** Unpaired *t* test, *N =* 3/group. **: *p* < 0.01. (**D)** Mann–Whitney test, *N* (WT) = 142 cells from 2 mice, *N* (KO) = 83 cells from 1 mouse. ****: *p* < 0.0001. (**E)** Scale: 2 μm, in inserts: 1 μm. (**F, G)** Scale: 25 μm; Mann–Whitney test, *N* (WT) = 162 cells from 3 mice, *N* (KO) = 281 cells from 5 mice. **: *p* < 0.01, ***: *p* < 0.001, ****: *p* < 0.0001. Data underlying this figure can be found in S1 Data. DIV, days in vitro; E17; embryonic day 17 KO, knockout; ns, not significant; TMX, tamoxifen; WT, wild type.

Available RNA-seq data as well as our results (Fig 1E) demonstrate that the *Slc12a2* gene is expressed by microglia [21,40]. In line with this, our further qPCR data revealed that mRNA levels of NKCC1 in microglial cells isolated from newborn (1.052 ± 0.054) or adult (0.89 ± 0.098) C57BL/6J mice are comparable to those in neural progenitors derived from embryonic brains (1.30 ± 0.05; Fig 2A). However, NKCC1 mRNA expression markedly decreased when cells were grown in culture for 10 days (Fig 2A), making it difficult to study NKCC1 function in microglia using in vitro techniques (see also [58]).

To study the functional role of NKCC1 in microglia in vivo, we generated a novel transgenic mouse line in which microglial NKCC1 was deleted (Fig 2B). *Slc12a2* expression in isolated microglia derived from tamoxifen-inducible microglial NKCC1 knockout (KO) mice was markedly reduced in comparison with wild-type (WT) littermates (WT: 1.46 ± 0.11, KO: 0.50 ± 0.016; Fig 2C) as assessed by qPCR. Reduced NKCC1 protein expression was also confirmed by unbiased fluorescent density measurements after systematic random sampling of a great number of individual microglial cells from Iba1/NKCC1 stained slices. In fact, a discrete NKCC1 labeling was seen in the plasma membrane of microglial cells in WT mice, which was absent in microglial NKCC1 KO mice (Fig 2D and 2E).

Microglia are known to respond to physiological and pathological challenges with fast morphological transformation [47], while the role of NKCC1 in cell volume regulation has been highlighted in previous studies concerning other cell types [48,49]. To study the role of NKCC1 in microglial cell shape, we performed automated morphological analysis using perfusion-fixed brain sections obtained from WT and microglial NKCC1 KO mice (Fig 2F and 2G). First, we aimed to test whether microglial NKCC1 deficiency has any impact on baseline cell morphology. Our data revealed that NKCC1-deficient microglia displayed a 23.8% higher cell surface, 19% higher branch volume, and 33.5% more branches compared to WT cells (Fig 2F and 2G), while we observed no changes in the cell body volume of KO versus WT microglia (Fig 2G). According to these data, microglial NKCC1 is likely to be involved in shaping baseline cell morphology.

### Microglial process motility is altered by central NKCC1 inhibition

**Fig 3:**
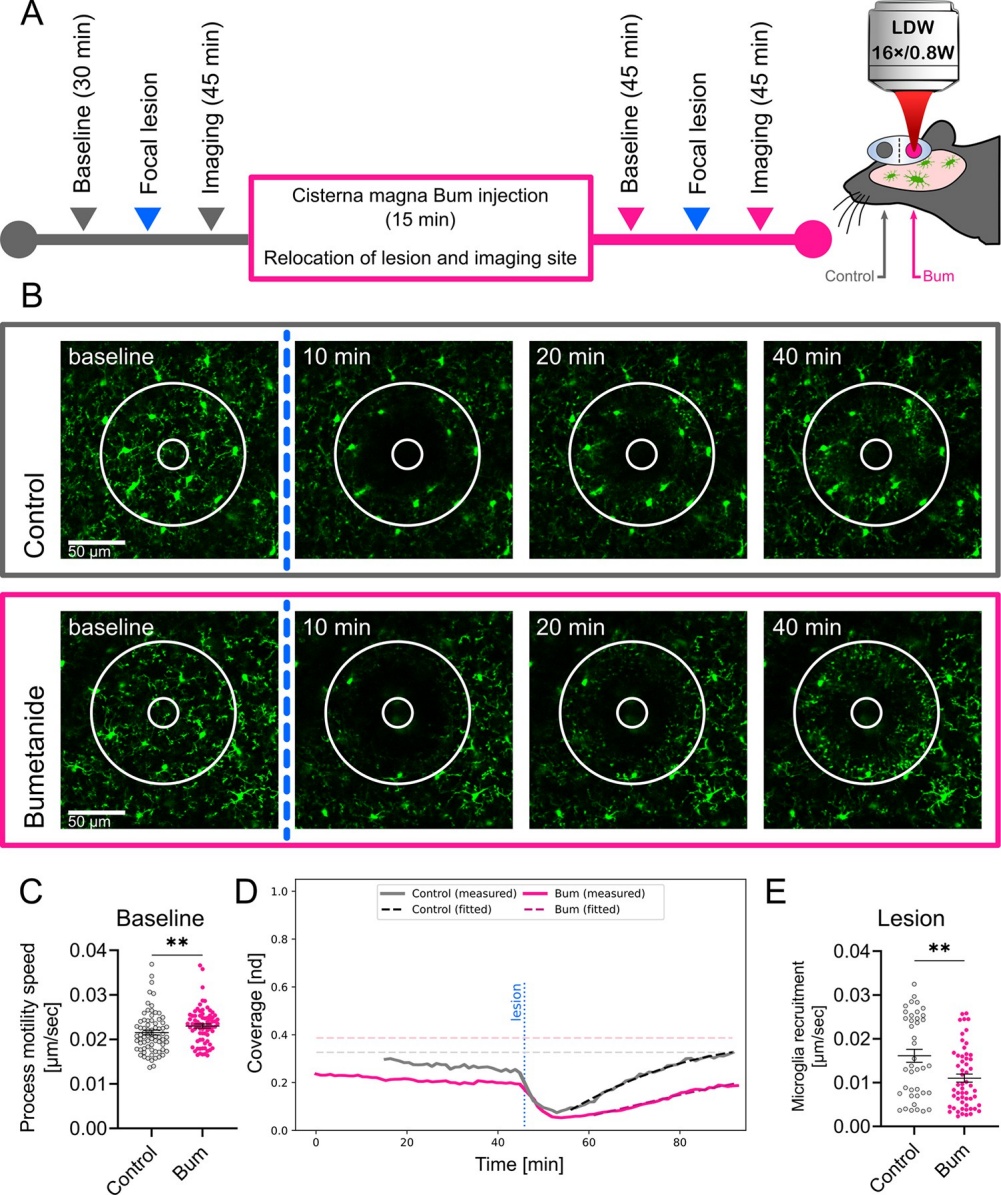
Microglial process dynamics and injury-induced process recruitment are altered by bumetanide. **(A)** Outline of the 2-photon imaging experiment performed in Cx3CR1^+/GFP^ (microglia reporter) mice. Imaging was performed to assess baseline microglial process motility and response to laser-induced injury followed by Bum administration into the cisterna magna and identical measurements at a different, undisturbed cortical region. (**B)** Representative images of microglial responses at selected time points, taken from S1 Video. The lesion site is marked with a circle that completely covers the area of the lesion. The ablation zone (i.e., the inner circle) was excluded from the analysis (see Materials and methods). (**C)** Mean base-state velocity distribution of processes from manual image tracking. Data show a 7% higher mean velocity of cell processes after Bum administration. (**D)** Evaluation of microglial process coverage over time in the lesion-centered circular area generated by a custom image analysis pipeline. Solid lines show the proportion of the area covered by microglial GFP signal. Dashed curves show the calculated values of coverage from the best-fitting curves. Horizontal lines are the predicted maximal coverage values for the lesion site; vertical line is the time point of the lesioning. (**E)** Calculated postlesion velocities of microglial process recruitment using automated image analysis followed by model fitting. Data show a significant decrease (31.7%) in mean frontline velocity of cells with Bum administration. (**B)** Scale: 50 μm. (**C)** Mann–Whitney test, N_Control_ = 73, N_Bum_ = 76 processes from 5 mice, **: *p* < 0.01. (**E)** Mann–Whitney test, N_Control_ = 40, N_Bum_ = 56 fitted values from 5 and 7 mice, respectively, **: *p* < 0.01. Data underlying this figure can be found in S1 Data. Bum, bumetanide.

To assess whether NKCC1 is involved in the regulation of dynamic microglial process surveillance and injury-induced microglial responses, we performed in vivo 2-photon imaging in microglia reporter Cx3CR1^+/GFP^ mice in combination with bumetanide treatment (Fig 3). We tracked prelesion (baseline) process motility followed by focal laser-induced lesioning, which was repeated after bumetanide administration into the cisterna magna in a different (undisturbed) part of the cerebral cortex in the same animals (Fig 3A and 3B). The cisterna magna application protocol had been extensively tested in previous studies for effective drug penetration into the brain parenchyma and direct actions on microglia by using PSB0739, a selective P2Y12R antagonist [31]. Bumetanide caused a small but observable (7%) increase in the mean velocity of microglial processes (Fig 3C). In contrast, lesion-induced recruitment of microglial processes showed a marked, 31.7% reduction after bumetanide treatment (*N =* 7) (Fig 3D and 3E and see **S1 Video**). These experiments indicate that beyond shaping cell morphology, microglial NKCC1 also regulates dynamic microglial actions both under physiological and pathological conditions.

### The absence of microglial NKCC1 induces NLRP3 and potentiates inflammatory cytokine production in the cerebral cortex

**Fig 4:**
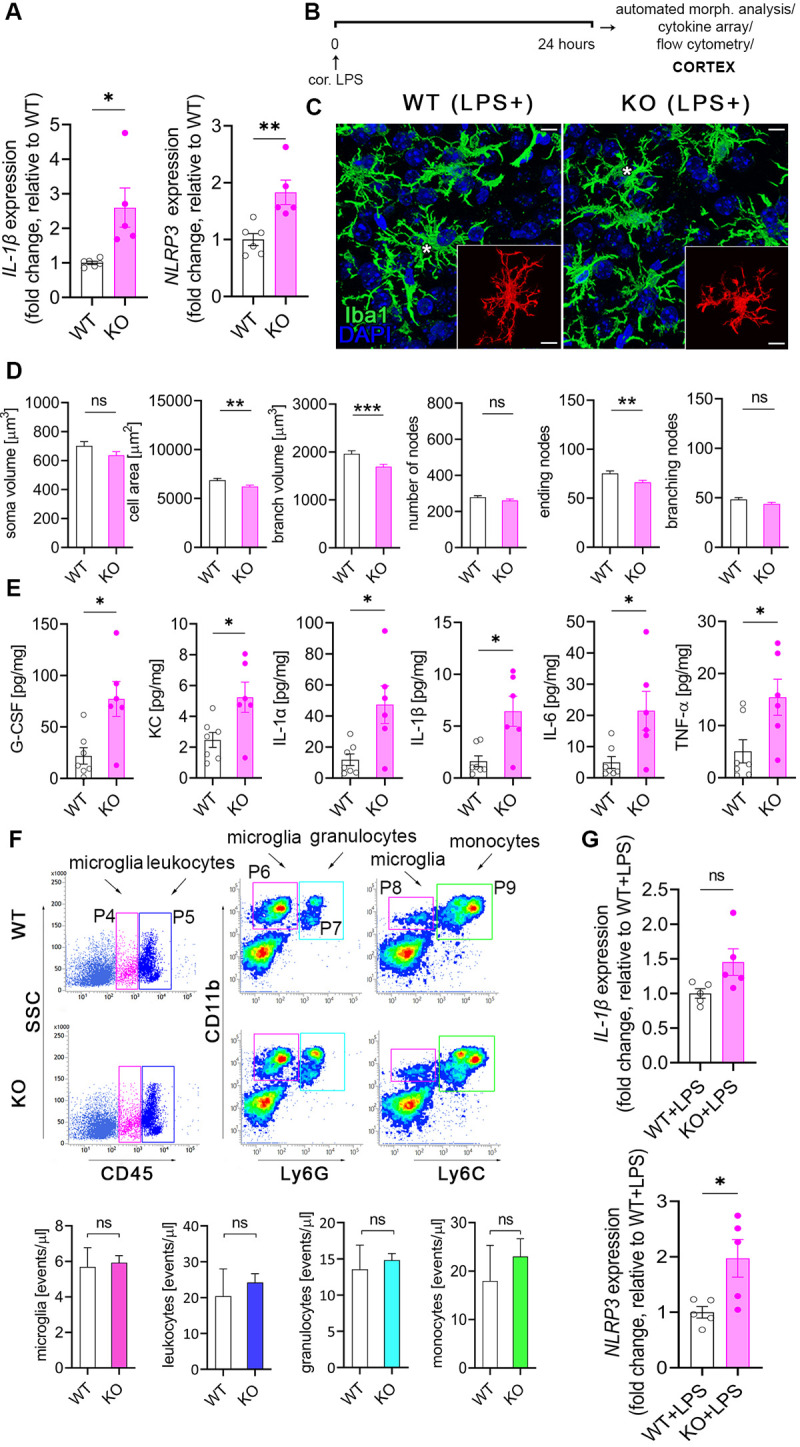
The absence of microglial NKCC1 boosts inflammatory cytokine production in the cerebral cortex in response to an inflammatory stimulus. **(A)** Baseline NLRP3 and IL-1β mRNA expression is increased in isolated NKCC1 KO microglia compared to WT cells. (**B)** Experimental outline of automated morphological analysis, cytokine array, and flow cytometry. **(C, D)** Automated morphological analysis shows that activated NKCC1-deficient microglia are slightly smaller than their WT counterparts. **(E)** LPS-induced cytokine levels are significantly higher in the cortices of microglial NKCC1 KO mice than in WT. (**F)** Flow cytometric dot plots show that microglial NKCC1 deficiency does not alter the number of CD11b^+^, CD45^int^ microglia (P4 gate) or numbers of infiltrating CD11b^+^, CD45^high^ leukocytes (P5 gate), CD11b^+^, Ly6C^high^ monocytes (P9 gate) and CD11b^+^, Ly6G^high^ granulocytes (P7 gate) in response to intracortical LPS administration. See corresponding data on peripheral cytokine levels and immune cell populations in S3 Fig**. (G)** Increased NLRP3 and IL-1β mRNA levels are sustained in NKCC1 KO and WT microglia 24 hours after intracisternal LPS administration**. (A)** Unpaired *t* test; *p < 0.05, **p < 0.01; *N* (WT) = 6, *N* (KO) = 5. (**C)** Scale: 25 μm. (**D)** Mann–Whitney test, *N* (WT) = 108 cells from 6 mice, *N* (KO) = 92 cells from 4 mice, **p < 0.01, ***p < 0.001. (**E)** Unpaired *t* test, *: *p* < 0.05; *N* (WT) = 7, *N* (KO) = 6. (F) Unpaired *t* test, *N* (WT) = 4, *N* (KO) = 4. (G) Unpaired *t* test; *p < 0.05; *N* (WT + LPS) = 5, *N* (KO + LPS) = 5. Data underlying this figure can be found in S1 Data. KO, knockout; ns, not significant; WT, wild type.

To assess the effect of NKCC1 deletion on microglial inflammatory states and responses, we first examined the expression of IL-1β and NLRP3. Assembly of the NLRP3 inflammasome is known to be a key step for processing of pro-IL-1β by caspase-1, and the release of mature IL-1β from microglia or macrophages [50,51]. qPCR data revealed elevated expression of both IL-1β and NLRP3 in NKCC1 KO microglia even in the absence of inflammatory stimulus (Fig 4A). Next, we examined the impact of NKCC1 deficiency on morphological characteristics of microglia after activation by endotoxin that primes microglial inflammatory responses and leads to morphological transformation [52]. Unbiased, automated morphological analysis showed that both WT and NKCC1 KO microglia respond to intracortical LPS administration with dramatic morphological conversion (Fig 4B and 4C). However, NKCC1-deficient microglia were significantly smaller than their WT counterparts, displaying a 10% smaller cell surface, 12.5% smaller cell volume, and 13.7% smaller branch volume (Fig 4D). According to these data, microglial NKCC1 is likely to be involved in shaping cell morphology and regulation of cell volume after inflammatory challenges.

To test the impact of microglial NKCC1 deletion on the production of central inflammatory mediators, we examined LPS-induced cytokines and chemokines in microglial NKCC1 KO and WT mice 24 hours after intracortical LPS injections in brain homogenates (Fig 4E). Importantly, deletion of microglial NKCC1 markedly potentiated LPS-induced levels of G-CSF (3.53-fold), KC (2.12-fold), IL-1α (3.98-fold), IL-1β (3.96-fold), IL-6 (4.35-fold), and TNF-α (3-fold) in the cerebral cortex compared to controls (Fig 4E). In contrast, intracortical LPS treatment did not alter cytokine levels or T cell, B cell, monocyte, or granulocyte numbers in the spleen or liver (S3A–S3C Fig). Similarly to that seen after intracortical pharmacological inhibition of NKCC1 by bumetanide, neither the number of CD11b^+^ CD45^int^ microglial cells nor blood-borne CD11b^+^ CD45^high^ leukocytes, CD11b^+^ Ly6C^high^ monocytes, or CD11b^+^ Ly6G^high^ granulocytes were altered as a result of microglial NKCC1 deficiency (Fig 4F). We also tested whether increased baseline IL-1β and NLRP3 mRNA levels seen in NKCC1 KO microglia (Fig 4A) were maintained in these cells 24 hours after intracisternal LPS administration. We found that significantly increased NLRP3 mRNA levels (by 100%) were still present in NKCC1 KO microglia isolated from the brain by using magnetic separation, while a strong trend (*p* = 0.057) to increased IL-1β mRNA by 50% was also observed (Fig 4G). These findings corroborated our previous results on cortical NKCC1 blockade by bumetanide, confirming that the inhibition of microglial NKCC1 markedly potentiates central cytokine production upon inflammatory challenges, with a likely role for the NLRP3 inflammasome in the excessive production of IL-1β.

### Deletion of NKCC1 from microglia results in altered membrane currents and a hyperpolarizing shift in the reversal of swelling-induced current

**Fig 5:**
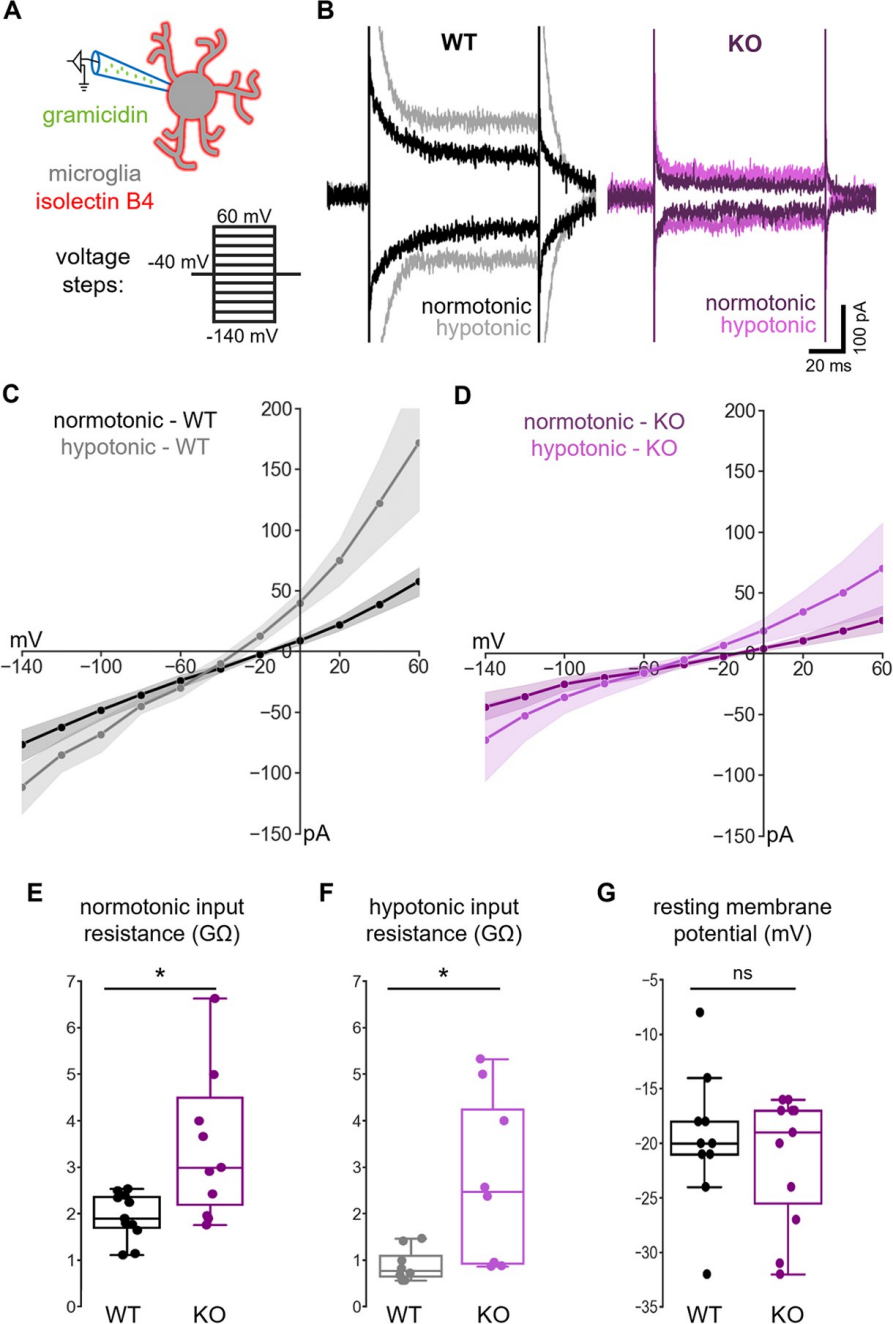
Deletion of NKCC1 from microglia results in altered membrane currents. **(A)** Schematic representation of the experiment. Perforated patch-clamp recordings were performed on isolectin B4–labeled microglial cells in acute hippocampal slice preparations. Current responses to a train of voltage steps from −140 to 60 mV with 20 mV increments and a duration of 100 ms were measured in voltage-clamp mode (holding potential: −40 mV) both in normotonic, and after 5-minute perfusion with hypotonic ACSF (50% dilution). (**B)** Example traces of recordings from WT (black: normotonic, gray: hypotonic ACSF) and NKCC1 KO (purple: normotonic, violet: hypotonic ACSF) animal. Traces show responses at −140 and +60 mV voltage steps in both conditions. (**C)** Average I-V curve responses from WT in normotonic (*N =* 11 cells, black with SEM) and in hypotonic (*N* = 8 cells, gray with SEM) condition. (**D)** Average I-V curves from NKCC1 KO in normotonic (*N* = 11 cells, purple with SEM) and in hypotonic (*N* = 8 cells, violet with SEM) condition. (**E)** Input resistance of WT versus KO cells in normotonic condition. (**F)** Input resistance of WT versus KO cells in hypotonic condition. (**G)** Resting membrane potential in normotonic condition of WT versus NKCC1 KO microglial cells measured in current-clamp mode (0 pA injected current). (**E)** Mann–Whitney; *N* (WT) = 11 cells from 8 animals, *N* (KO) = 11 cells from 7 animals; *: *p* < 0.05. (**F)** Mann–Whitney; *N* (WT) = 8 cells from 6 animals, *N* (KO) = 8 cells from 5 animals; *: *p* < 0.05. (**G)** Mann–Whitney; *N* (WT) = 10 cells from 8 animals, *N* (KO) = 11 cells from 7 animals. Data underlying this figure can be found in S1 Data. KO, knockout; ns, not significant; WT, wild type.

To study the consequences of NKCC1 deletion on microglial ion regulation and membrane currents, we made perforated patch-clamp recordings on native microglia in acute hippocampal slice preparations from WT and microglial NKCC1 KO mice. With this method, the intracellular Cl^−^ concentration remains unaffected by the pipette solution. Recordings were done in voltage-clamp mode at a holding potential of −40 mV, while voltage steps (−140 mV to +60 mV with 20 mV increments and 100 ms duration) were delivered to construct I-V plots for individual cells (Fig 5A). An exposure to hypotonic ACSF solution (50% dilution, 5 minutes) was used to identify the outwardly rectifying current in response to cellular swelling, which is considered to be mainly mediated by the largely Cl^−^ permeable volume-regulated anion channels (VRACs) [53–55]. Our results showed that current responses under normotonic and hypotonic conditions (Fig 5B) were qualitatively similar to those published previously regarding WT microglia in acute slice preparations [56]. I-V curves (Fig 5C and 5D) indicated a much higher resistance of the KO versus WT cells, especially at more positive voltage levels under both normotonia and hypotonia. The slope of I-V curves between the −40 mV and 0 mV voltage steps (Fig 5E and 5F) showed significantly higher input resistance of KO microglia both in normotonia (WT: 1.91 GΩ, q1: 1.71, q3: 2.38 versus KO: 3.00 GΩ, q1: 2.20, q3: 4.50; Mann–Whitney, *p* < 0.05) and hypotonia (WT: 0.78 GΩ, q1: 0.66, q3: 1.11 versus KO: 2.47 GΩ, q1: 0.94, q3: 4.25; Mann–Whitney, *p* < 0.05). We also measured resting membrane potential of microglia (Fig 5G) where no statistically significant difference was observed between WT and KO (WT: −20 mV, q1: −21, q3: −18 versus KO: −19 mV, q1: −26, q3: −17; Mann–Whitney, not significant). In order to compare the responses of WT and KO microglial cells to cell swelling, we isolated the swelling-induced current component by subtracting the currents obtained at each point of voltage in normotonic conditions from those recorded during the hypotonic response (S4A Fig). The resulting I-V plot of the swelling-induced currents showed a reversal potential (E_swell_−a rough estimate of the Cl^−^ equilibrium potential, E_Cl-_), which was more negative in the KO cells (WT: −47 mV, q1: −53, q3: −38 versus KO: −68 mV, q1: −91, q3: −46; Mann–Whitney, *p* < 0.05; S4B Fig). Because of the variation of the individual data points related to reversal and resting potentials, we calculated the driving force (DF = V_rest_ − E_swell_) for each WT and KO cell (S4B Fig), which provides a more direct parameter of the efficacy of ion regulation than ionic reversal potentials [1]. We found significantly higher DF values in the case of NKCC1 KO versus WT microglia (WT: 28 mV, q1: 21, q3: 33 versus KO: 50 mV, q1: 30, q3: 65; Mann–Whitney, *p* < 0.05), supporting the view that the lack of microglial NKCC1 results in a lower intracellular Cl^−^ concentration.

To investigate the potential compensatory mechanisms that may arise in response to NKCC1 deletion in microglial cells, we evaluated the expression of some relevant genes that are reportedly expressed by microglia and are known to regulate ion concentrations, membrane potential or response to osmotic stress (S5 Fig; [57,58]). *Slc9a1*, *Slc8a1*, *Slc12a6*, *Clic1*, *Clcn3*, *Kcnk13*, *Kcnk6*, *Kcnj2*, *Kcna3*, and *Sgk1* mRNAs did not show altered expression between WT and NKCC1 KO microglia (S5A and S5C Fig), but we found a more than 2-fold increase in *Lrrc8d*, the D subunit of VRAC in response to NKCC1 deletion (S5A Fig). Importantly, *Lrrc8d* (VRAC) is involved in increasing the permeability for a broad range of uncharged organic osmolytes [59]. Interestingly, intracisternal LPS treatment resulted in a marked reduction of NKCC1 and P2Y12R mRNA levels (Fig 1E), in line with the down-regulation of *Slc9a1*, *Slc8a1*, *Lrrc8d*, *Clic1*, *Clcn3*, *Kcnk13*, *Kcnk6*, *Kcnj2*, *Sgk1* genes (S5B Fig) of which *Slc9a1*, *Clic1*, *Kcna3*, *Kcnj2*, *Kcnk13* genes are known to play a role in maintaining ramified morphology and resting state under physiological conditions [35,39,58,60].

### Deletion of microglial NKCC1 increases infarct volume, brain edema and worsens neurological outcome after MCAo

**Fig 6:**
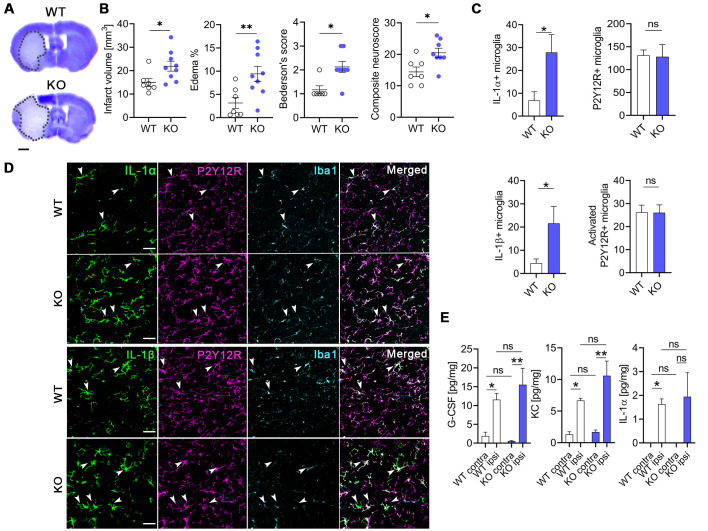
Deletion of microglial NKCC1 increases infarct volume, brain edema, and worsens neurological outcome after MCAo. **(A, B)** Microglial NKCC1-deficient mice (KO) show larger infarct volume as assessed on cresyl violet-stained brain sections and more severe neurological outcome compared to WT mice. (**C, D)** Microglial NKCC1 deletion results in higher levels of IL-1α and IL-1β 24 hours after MCAo. **(E)** Cytokine levels in the cortex do not differ 8 hours after MCAo in KO mice compared to WT. **(A)** Scale: 1 mm. **(B)** Unpaired *t* test, *N* (WT) = 7, *N* (KO) = 9; *: *p* < 0.05, **: *p* < 0.01. **(C)** Mann–Whitney test, *: *p* < 0.05; *N* (WT) = 7, *N* (KO) = 8. (**D)** Scale: 50 μm. (**E)** Kruskall–Wallis test followed by Dunn’s multiple comparison test; *N =* 6/group. *: *p* < 0.05, **: *p* < 0.01. Data underlying this figure can be found in S1 Data. KO, knockout; MCAo, middle cerebral artery occlusion; ns, not significant; WT, wild type.

Because Na^+^-coupled Cl^−^ importers and their upstream regulatory serine–threonine kinases (WNK-SPAK-OSR1) are involved in maintaining intracellular ionic homeostasis as well as regulation of cell volume [2,49], inhibiting these cotransporters is a subject of interest in ischemic stroke therapy and in other forms of acute brain injury (brain trauma, SAH) where cerebral edema is a major contributor to poor clinical outcome [2,49]. Thus, we investigated whether microglial NKCC1 deficiency influences the severity of brain injury after experimental stroke in microglial NKCC1 KO mice subjected to transient, 45-minute long middle cerebral artery occlusion (MCAo). Twenty-four hours after reperfusion, elimination of microglial NKCC1 was not associated with obvious alterations in astroglial GFAP levels or AQP4 levels in perivascular astrocyte endfeet (S6 Fig). However, we observed a significant increase in brain edema, which was accompanied by a 47% increase in lesion volume (Fig 6A and 6B; *p* = 0.0262) and poor neurological outcome in NKCC1 KO mice (Fig 6B; Bederson’s score: WT: 1.2 ± 0.2, KO: 2.1 ± 0.2; composite neurological score: WT: 14.4 ± 1.5, KO: 20.5 ± 1.5). Furthermore, experimental stroke induced a 4-fold up-regulation of IL-1α and 4.8-fold increase in IL-1β expressing NKCC1 KO microglia cells compared to WT animals, while the overall microglia density and the number of activated microglia were not different at 24-hour reperfusion (Fig 6C and 6D). We also assessed whether increases in IL-1 production take place early after brain injury before the majority of the infarct is formed. Cytometric bead array did not reveal altered cytokine production in cortical homogenates shortly (8 hours) after MCAo (Fig 6E). Taken together, these results indicate that microglial NKCC1 deficiency results in augmented brain inflammation, brain edema, and increased brain injury after experimental stroke.

## Discussion

To our knowledge, we show for the first time that NKCC1 is functionally active in microglia in the adult mouse brain and that microglial NKCC1 is involved in shaping both baseline and reactive microglia morphology and responses. We present evidence that NKCC1 regulates microglial membrane conductance and adaptation to swelling-induced volume changes in a cell-autonomous manner, while the lack of NKCC1 results in primed microglial inflammatory states as evidenced by elevated NLRP3 and IL-1β levels. In line with this, while systemic NKCC1 blockade attenuates LPS-induced inflammation in the brain, central pharmacological inhibition or genetic deletion of microglial NKCC1 potentiates inflammatory cytokine production. Microglial NKCC1 deletion also increases brain injury, inflammation, and cerebral edema and leads to worse neurological outcome after acute brain injury induced by experimental stroke. Thus, microglial NKCC1 emerges as an important regulator of central inflammatory responses.

As pointed out in the Introduction, there is little direct information on whether systemically applied bumetanide might exert therapeutic effects by directly blocking NKCC1 in the brain and, particularly, in central neurons. To unravel the effects of pharmacological inhibition of NKCC1 in the periphery and the brain, we investigated the systemic versus central effects of bumetanide in response to bacterial endotoxin (LPS) administration, which is widely used to trigger inflammatory cytokine responses [41,52]. Intraperitoneally injected bumetanide reduced intracortical LPS-induced proinflammatory cytokine/chemokine responses in the brain, similarly to that found in the case of LPS-induced lung injury attributed to NKCC1-mediated macrophage activation [61]. These findings are also consistent with the suggested beneficial therapeutic effects of systemic NKCC1 blockade in CNS pathologies, which are associated with inflammation [1,7,8,10–12,62].

Importantly, intracortical administration of bumetanide had exactly the opposite effect on intracortical LPS-induced cytokine production to that seen after systemic bumetanide treatment, resulting in elevated cytokine levels in the cortex. These markedly different outcomes clearly demonstrate the difficulty to interpret pharmacological interventions in the absence of data on the precise cellular targets of NKCC1 actions in the brain and highlight the need for cell-specific NKCC1 targeting studies.

Because microglia are the key source of inflammatory cytokines in the brain, we tested whether microglial activity could be directly related to NKCC1 function. Indeed, we observed comparable *Slc12a2* expression in microglia, isolated from either newborn or adult mouse brains, to that seen in neural progenitors. We also revealed NKCC1 protein in cortical microglial cells, which is in line with recent observations showing microglial NKCC1 expression in the superficial spinal dorsal horn in rats [63], and also supported by single-cell transcriptomic studies [21,40]. Further on, we demonstrated that NKCC1 levels are markedly down-regulated in microglia in response to LPS, alongside with decreased P2Y12R levels (a purinergic receptor regulating microglial cell–cell interactions under both physiological conditions and in response to injury [21,44,64–66]), suggesting that impaired microglial NKCC1 function alters microglial responses to injury or inflammatory stimuli.

To investigate the cell-autonomous effects of NKCC1 in microglia, we generated a new, microglial NKCC1 KO transgenic mouse line. We found that the absence of NKCC1 resulted in a more branching microglial morphology in resting state and a more amoeboid shape under inflammatory conditions. These results paralleled enhanced baseline process motility in the intact brain, in vivo, and significantly reduced process recruitment to the site of the acute lesion, when microglial NKCC1 was blocked by intracisternal bumetanide during 2-photon imaging. While the use of bumetanide was required for in vivo imaging of microglia due to the absence of microglial reporter protein expression in microglial NKCC1 KO mice, the cisterna magna application protocol had been previously shown to effectively target parenchymal microglia by using PSB0739, a selective P2Y12R antagonist [31]. As centrally administered bumetanide may also reach other NKCC1-expressing cells (e.g., neurons and astrocytes), we cannot exclude the possibility that microglial responses might have been influenced indirectly by other cell types in this experiment. Nevertheless, the net effect of central NKCC1 blockade by bumetanide leads to markedly impaired microglial process response to acute injury. Associations of NKCC1 with actin cytoskeleton dynamics [67,68] are well documented, which take place via controlling F-actin organization through Cofilin-1 and RhoGTPase activity [67]. Collectively, our results suggest that regulation of targeted process outgrowth in microglia is NKCC1 dependent, in addition to its known dependency on microglial P2Y12R, while THIK-1, a potassium channel that regulates baseline microglial surveillance and process dynamics, is not required for this process [35].

Importantly, we also found that the absence of microglial NKCC1 renders microglia to express higher levels of NLRP3 and IL-1β mRNA even without any exposure to inflammatory stimuli. It is well established that changes in microglial membrane potential and ion currents markedly determine cell volume regulation, process dynamics, and inflammatory responses of microglia. For example, depolarization is associated with reduced branching in microglia [35,69], while potassium or chloride efflux are essential for NEK7-NLRP3 interaction during the assembly of the NLRP3 inflammasome [38,50], which is required for the production of mature IL-1β [37,38,50]. The regulation of these processes appears to be highly complex. The TWIK2 two-pore domain K^+^ channel (K_2P_) mediates K^+^ efflux triggering NLRP3 activation in lung macrophages [37], while THIK-1 has been identified as the main K^+^ channel in microglia [35], although RNA profiling [21,40] also revealed high expression of TWIK2 in this cell type. In line with this, blocking THIK-1 function inhibits the release of IL-1β from activated microglia, consistent with potassium efflux being necessary for inflammasome activation [35].

To reveal how microglial NKCC1 may contribute to these processes, we investigated whether deletion of NKCC1 leads to altered expression of ion channels or transporters that have been linked with microglial ion- and volume regulation and with inflammatory cytokine production (see references in S4D Fig). We could not observe changes in THIK-1 or TWIK2 mRNA levels by qPCR, but the subunit D of VRAC was up-regulated in KO animals. Subunit D is involved in the transport of uncharged organic osmolytes by VRAC, and it may be important for volume reduction without a major release of inorganic ions [59] for a cell coping with potentially lowered inorganic ion levels due to NKCC1 loss. Changes in the expression of other VRAC subunits may also be important for microglial inflammatory processes. For example, LRRC8A-containing anion channels are known associate with NADPH oxidase 1 (Nox1) and regulate superoxide production and TNF-α signaling in vascular smooth muscle cells [59,70]. Thus, changes in microglial ROS production in the absence of NKCC1 will need to be investigated in future studies.

To examine the effects of NKCC1 deletion on microglial membrane properties, we conducted perforated patch recordings in hippocampal slices from WT and KO animals. Importantly, we found that NKCC1 KO cells had an input resistance almost twice as high as that measured in WT under both normotonia and hypotonia. We also characterized the swelling-induced current for WT and KO microglia, which is considered to be mainly mediated by the largely Cl^−^ permeable VRACs [53–55]. In agreement with an expected decrease in cellular Cl^−^ uptake upon NKCC1 deletion [1], E_swell_ was significantly more negative in the KO versus WT microglia, which was further supported by comparing the driving forces of swelling-induced currents in individual cells, yielding higher values in KO cells. However, the driving force at V_rest_ had a positive polarity (outward current) in all cells of both genotypes, which indicates that in addition to NKCC1 and VRAC, an active Cl^-^ extruder is likely to be present in microglia. While the identity of the latter cannot be deduced from the present data, there is evidence that K^+^- Cl^-^ cotransporter (KCC) isoforms, such as the volume-sensitive KCC1, are expressed in microglia [71]. It is worth noting here that in numerous cell types, NKCC1 and KCCs are simultaneously involved in chloride regulation [72]. Our data indicate that the increased input resistance in KO was also paralleled with a decrease in the swelling-induced current. We have no mechanistic explanation for these two effects, but they are likely to reflect adaptations of microglia to genetic NKCC1 deletion with compensatory changes not revealed by our qPCR studies. Such a coordinated adaptive response could involve down-regulation of VRACs and/or K^+^ channels among others. Upregulation of VRAC subunit D in NKCC1 KO microglia could represent a compensatory response to impaired VRAC function, as suggested by the reduced swelling-related conductance in these cells.

We also found that in line with increased NLRP3 and IL-1β mRNA levels in NKCC1 KO microglia, LPS stimulation resulted in elevated NLRP3 mRNA levels even after 24 hours upon LPS treatment and potentiated the production of IL-1β and other cytokines in the cortex of microglial NKCC1 KO mice compared to control mice. Importantly, deletion of microglial NKCC1 had an identical effect on inflammatory cytokine levels to that seen after NKCC1 inhibition by bumetanide, suggesting that central bumetanide actions involve blockade of microglial NKCC1. In line with this, we could not find significant changes in two key astroglial markers, GFAP and AQP4, after central bumetanide application in response to LPS treatment, arguing against a major role of astroglial NKCC1 in the observed differences on the timescale of the present study. While a role for astroglial NKCC1 in mediating central bumetanide actions has been previously reported [18,19], our present work shows that central bumetanide effects involve a substantial microglial component via NKCC1.

Proinflammatory cytokine production, particularly the effects mediated by IL-1, are known to influence acute brain injury induced by brain trauma or stroke [73–75]. We found significantly increased infarct volume (by 47%) in microglial NKCC1 KO mice after experimental stroke comparable to a striking, 60% increase in infarct size caused by highly efficient, selective microglia elimination [32]. Microglia depletion resulted in dysregulated neuronal network activity, an effect that is likely to be mediated via elimination of protective microglia–neuron interactions at somatic purinergic junctions [31]. It remains to be investigated in future studies, whether microglia–neuron interactions are altered in microglial NKCC1 KO mice, which could shape neuronal injury via diverse mechanisms related to changes in neuronal excitability [29,30,32,33]. In line with this, the absence of microglial NKCC1 may have contributed to increased neuronal injury via increased IL-1 production, as suggested by the established pathophysiological role of IL-1–mediated actions in different forms of brain injury [73,76].

In our experimental stroke studies, we could not detect increased cytokine production in the absence of microglial NKCC1 early (8 hours) after MCAo, while both IL-1α and IL-1β expression were markedly up-regulated in NKCC1 KO microglia 24 hours after reperfusion. Following stroke, impaired cell volume regulation results in cytotoxic cell swelling and edema formation, which peaks beyond the first 24 hours in both patients and experimental animals [77,78]. Cerebral edema was shown to be associated with increased phosphorylation of the SPAK/OSR1 kinases playing a key role in NKCC1 activation in various neural cell types [2,48,49] and a SPAK kinase inhibitor, ZT-1a, attenuated cerebral edema after stroke [79]. In line with this, NKCC1 is involved in homeostatic cell volume regulation [48,49], and bumetanide attenuates edema formation in response to ischemic or traumatic brain injury [2]. In light of these findings, our data showing impaired volume regulation and exaggerated cytokine production in NKCC1 KO microglia have a potentially high pathophysiological and clinical relevance.

In conclusion, we show that microglial NKCC1 plays a previously unrecognized role in shaping microglial phenotype and inflammatory responses. Strikingly, bumetanide-induced enhancement of neuroinflammation was mimicked by conditional deletion of microglial NKCC1, suggesting that microglial NKCC1 is a significant target of NKCC1 blockers, given that brain tissue levels of such drugs are sufficiently high [10,13,14]. Our results implying microglial NKCC1 in inflammation, brain edema formation, and responses to injury suggest that microglial NKCC1 actions should be considered when studying the effects of NKCC1 inhibitors in different CNS functions and pathologies. This is further emphasized by the broad clinical interest in pharmacological NKCC1 blockade. The present results also call for a reevaluation of the pharmacological effects of bumetanide in brain diseases with a significant inflammatory component.

## Materials and methods

### Experimental animals

Experiments were performed on adult male C57BL/6J (RRID: IMSR JAX:000664), NKCC1^fl/fl^, and NKCC1^fl/fl ΔCx3CR1^ (microglial NKCC1 KO) mice (all on C57BL/6J background). For experiments using LPS treatment P90-110 (postnatal day 90-110), and for MCAo experiments, P70-90 day-old mice were used. NKCC1^fl/fl^ mice were provided by Dr. Christian A. Hübner of University Hospital Jena of Friedrich Schiller University, Jena, Germany. Ubiquitous NKCC1 KO mice (P15-20, NKCC1 full KO, NKCC1^−/−^), generated by crossing C57BL/6NTac-Gt(ROSA)26Sor^tm16(cre)Arte^ and NKCC1^fl/fl^ mice, were used as immunostaining controls and were not subjected to other experimental procedures. Mice were housed in a 12-hour dark/light cycle environment, under controlled temperature and humidity and ad libitum access to food and water. All experimental procedures were in accordance with the guidelines set by the European Communities Council Directive (86/609 EEC) and the Hungarian Act of Animal Care and Experimentation (1998; XXVIII, Sect. 243/1998) and approved by the Animal Care and Experimentation Committee of the Institute of Experimental Medicine and the Government Office of Pest Country Department of Food Chain Safety, Veterinary Office, Plant Protection and Soil Conservation Budapest, Hungary under the number PE/EA/1021–7/2019 and Department of Food Chain Safety and Animal Health Directorate of Csongrád County Hungary. All experiments followed ARRIVE and IMPROVE guidelines [80,81]. A code was allocated to each animal using GraphPad’s random number generator and was randomly assigned to different treatment groups. During all surgical procedures and functional tests, the experimenter was blinded to the treatment.

### Generation and genotyping of the microglial NKCC1 knockout mouse line

Microglial NKCC1 deletion (NKCC1^fl/fl ΔCx3CR1^) was achieved by crossing tamoxifen-inducible B6.129P2(C)-CX3CR1tm2.1 (cre/ERT2)Jung/J mice (RRID:IMSR_JAX:020940JAX) [82] with the NKCC1^fl/fl^ mouse line [83], in which a region between exon 7 and exon 10 of *Slc12a2* gene was flanked with lox P sites. Microglial NKCC1 deletion was achieved by 2 intraperitoneally administered tamoxifen injections (100 μl of 20 mg/ml in corn oil, #T5648, Sigma-Aldrich) 48 hours apart in 3- to 4-week-old male mice, shortly after weaning. In all functional experiments, mice were studied more than 3 weeks after the last tamoxifen injection to avoid interference with Cre-dependent recombination in peripheral myeloid cells [82]. In the case of selective microglia isolation by magnetic cell sorting, we used animals exposed to tamoxifen for at least 2 weeks as Cre activity was shown to increase most dramatically within the first week after initiating treatment [84].

In all experiments, conditional mutant mice heterozygous for cre and homozygous for NKCC1 flox (^cre/+ fl/fl^), referred to as NKCC1 KO throughout the manuscript, were used. In all cases, littermate controls—referred to as WT—were used, which were negative for cre and homozygous for NKCC1 flox (^+/+ fl/fl^). Heterozygous, conditional mutant mice were viable, fertile, average in size, and did not display any gross physical or marked behavioral abnormalities. Genotypes for *Slc12a2* encoding NKCC1 were determined from tail biopsy samples by conventional PCR using the following primers: 5′-GCAATTAAGTTTGGAGGTTCCTT, 5′- CCAACAGTATGCAGACTCTC and 5′-CCAACAGTATGCAGACTCTC; product sizes: 200 bps for WT, 260 bps for floxed, and 460 bps for KO.

### Microglia cell preparations and cultures

Microglia cells from either newborn (P0-1, male or female) or adult (P40-55, male) C57BL/6J, NKCC1^fl/fl^ (WT), or NKCC1^fl/fl ΔCx3CR1^ (KO) mice were isolated by magnetic separation using anti-CD11b microbeads (Miltenyi Biotec, Germany), with slight modification of the protocol described by Otxoa-de-Amezaga [85]. After transcardial perfusion with ice-cold phosphate-buffered saline (PBS), the brain tissues (cortices and hippocampi) were enzymatically dissociated with Neural Tissue Dissociation Kit-P (#130-092-628; Miltenyi Biotec). Myelin was removed by MACS Myelin Removal Beads II (#130-096-733, Miltenyi Biotec), then cells in a single-cell suspension were magnetically labeled with MACS CD11b microbeads (#130-093-634, Miltenyi Biotec) and were separated using MS columns (#130-042-201, Miltenyi Biotec, Germany). Cells selected with CD11b microbeads were plated onto poly-L-lysine precoated 96-well or 386-well plates at 3 × 10^4^ cell/cm^2^ density and were cultured at 37°C in a 95% air/5% CO_2_ incubator in DMEM/Glutamax medium (#31966–021, Gibco) supplemented with 10% fetal bovine serum (FBS, #FB-1090, Biosera), 1% Pen/Strep (10,000 U/ml; #15140–122, ThermoFisher Scientific), and 10 nM macrophage colony-stimulating factor (M-CSF; #PMC2044, ThermoFisher Scientific) for 10 days.

### Embryonic neural progenitor cell preparations

Embryonic hippocampal cells were prepared from C57BL/6J mice on embryonic day 17. After aseptically removing the hippocampi from the skull, tissue was freed from meninges and incubated in 0.05% trypsin–EDTA (#T4549, Sigma-Aldrich) solution with 0.05% Dnase I (#DN25, Sigma-Aldrich) in PBS for 15 minutes at 37°C.

### Intracortical and intracisternal injections

P90-110 mice were deeply anesthetized with fentanyl (0.05 mg/kg) and mounted into a stereotaxic frame, then were subjected to either single saline or LPS (lipopolysaccharide from *Escherichia coli* O26:B6; 200 nl of 5 mg/ml, rate = 200 nl/10 minutes; #L8274, Sigma-Aldrich) injections using a glass micropipette [86]. The coordinates for the injection were anterior–posterior −2.5 mm, lateral +1.5 mm, and ventral −0.25 mm from the bregma. Bumetanide, a specific inhibitor of NKCC1 in the brain, was coinjected with LPS (50 μM; #3108, Tocris). At 24 hours, mice were transcardially perfused with saline, and approximately 0.5 × 0.5 × 0.5 cm sized tissue pieces from the center of each injected cortical region were cut off and collected for cytokine array and flow cytometric analysis. For tissue sectioning, mice were perfused with saline followed by 4% paraformaldehyde in PBS. For real-time quantitative PCR (qRT-PCR) experiments to assess the effect of NKCC1 deficiency on microglial expression of genes, which contribute to ion homeostasis, membrane potential, cell volume regulation, or inflammation, LPS (5 μg dissolved in ACSF) was administered into the cisterna magna in 5 μl final volume, using a glass capillary. At 24 hours, mice were transcardially perfused with saline followed by CD11b+ magnetic cell sorting.

### Systemic administration of LPS and bumetanide

Male adult NKCC1^fl/fl^ mice were injected intraperitoneally with saline, LPS (2 mg/kg; O26:B6, #L8274, Sigma-Aldrich) or LPS (2 mg/kg) and bumetanide (25 mg/kg; #3108, Tocris). Intraperitoneal bumetanide injections were repeated twice, the first one 15 minutes prior to LPS injection, the second one 1 hour after LPS administration. The double injection aimed to ensure that in the critical time window, we have effective concentrations of bumetanide in the circulation. At 24 hours, saline-perfused spleen and brain samples were collected for cytokine measurements or flow cytometric analysis.

### Cytokine measurement

The levels of inflammatory cytokines and chemokines were measured in spleen and brain samples. Sample processing and protein determination were performed as described previously [87]. Mice were transcardially perfused with saline prior to the collection of spleen and brain samples (ipsilateral to injections). Tissue samples were homogenized in TritonX-100 and protease inhibitor-containing (1:100, #539131, Calbiochem) Tris–HCl buffer (TBS (pH 7.4)) and centrifuged at 17,000 *g*, for 20 minutes at 4°C. Protein level was quantified for every sample using BCA Protein Assay Kit (#23225, ThermoFisher Scientific). Then, measured cytokine levels were normalized for total protein concentrations. The concentrations of cytokines and chemokines were determined by BD Cytometric Bead Array (CBA) using BD CBA Flex Sets (G-CSF: #560152, KC: #558340, IL-1α: #560157, IL-1β: #560232, IL-6: #558301, TNF-α: #558299, BD, Becton, Dickinson and Company) according to the manufacturer’s instructions. Samples were acquired using a BD FACSVerse flow cytometer (BD, Becton, Dickinson and Company), and the results were analyzed by FCAP Array software (BD, Becton, Dickinson and Company).

### Flow cytometric analysis of brain and spleen and liver samples

For flow cytometric analysis, cells were isolated from mouse brains with Collagenase D (0.5 mg/ml, #11088866001, Roche), DNase I (10 μg/ml, #DN25, Sigma-Aldrich) dissolved in 10% FBS containing DMEM (#6546, Sigma-Aldrich), then the cell suspension was passed through a 40-μm cell strainer (Corning). After enzymatic dissociation, the cells were resuspended in 40% Percoll solution and overlayed on 70% Percoll (#17-0891-01, GE Healthcare). After a density centrifugation step (at 2,100 rpm, 30 minutes), mononuclear cells were collected from the interphase of 40%/70% Percoll. Spleen and liver were mechanically homogenized, and red blood cells were removed by centrifugation. Before acquisition, brain, spleen, and liver cells were diluted with FACS buffer and were incubated with anti-mouse CD16/32 to block Fc receptor. Brain cells or spleen and liver leukocytes were incubated with cocktails of selected antibodies: T cells—anti-mouse CD8a-PE (1:200, #12-0081-82, eBioscience), anti-mouse CD3-APC clone 17A2 (1:200, #17-0032-80, eBioScience), anti-mouse CD4-FITC (1:200, #11-0043-82, eBioscience), anti-mouse CD45-PerCP/Cy5.5 (1:200, #103131, BioLegend); B cells/granulocytes—anti-mouse CD19-FITC (1:200, #11-0193-81, eBioScience), anti-mouse Ly-6C-PE-Cy7 (1:500, #25-5932-80, eBioScience), anti-mouse Ly-6G-APC (1:500, #127613, BioLegend); monocytes/granulocytes—anti-mouse CD11b-FITC (1:200, #11-0112-81, eBioscience); anti-mouse Ly-6C-PE-Cy7; anti-mouse Ly-6G-APC, CD45-PerCP/Cy5.5. To exclude dead cells, some coctails contained propidium iodide (3 μM; #P1304MP, ThermoFisher). Cells were acquired on a BD FACSVerse flow cytometer, and data were analyzed using FACSuite software (BD, Becton, Dickinson and Company). Cell counts were calculated by using 15 μm polystyrene microbeads (#18328–5, Polysciences).

### RNA isolation and cDNA reverse transcription

For qRT-PCR measurements, CD11b+ magnetic sorted microglia cells or embryonic neural progenitor cells were homogenized in QIAzol Lysis Reagent (#79306, QIAGEN) either immediately after isolation or after 10 days of in vitro culturing. Total RNA was isolated using Direct-zol RNA Miniprep Kits (#R2052, Zymo Research) following the manufacturer’s protocol. The RNA purity and concentration were assessed by NanoDrop ND-1000 spectrophotometer (Nanodrop Technologies). The isolated RNA was then stored at −80°C. RNA was subjected to DNase I (#AM2224, Ambion) treatment in the presence of RNase H inhibitor (#AM2682, Ambion). Standardized quantities of RNA were reverse transcribed to cDNA using the SuperScript II First-strand Reverse Transcriptase system (#18064014, ThermoFisher Scientific) and random hexamers (#48190011, Invitrogen) supplemented with RNase H inhibitor (#AM2682, Ambion).

### Real-time quantitative PCR (qRT-PCR)

qRT-PCR was performed with QuantStudio12K Flex qPCR instrument (Applied Biosystems), using TaqMan Gene Expression Assays and the TaqMan Gene Expression Master Mix (#4369016, ThermoFischer Scientific). All mouse TaqMan Gene Expression Assays used for amplification reactions were obtained from ThermoFischer Scientific: *Slc12a2* (Mm01265951_m1, targeting exon 1 to 10); (Mm00436546_m1, targeting exon 8 to 10, used for the validation of microglia-specific NKCC1 deletion; see Fig 2C), *Hprt* (Mm03024075_m1), *Slc12a6* (Mm01334052_m1), *Slc8a1* (Mm01232254_m1), *Slc9a1* (Mm00444270_m1), *Clcn3* (Mm01348786_m1), *Clic1* (Mm00446336_m1), *Kcnk6* (Mm01176312_g1), *Kcnj2* (Mm00434616_m1), *Kcna3* (Mm00434599_s1), *Lrrc8d* (Mm01207167_m1), *Sgk1* (Mm00441380_m1), *NLRP3* (Mm00840904_m1), *pro-IL-1β* (Mm00434228_m1). The amplification was performed under the following cycling conditions: 95°C for 10 minutes, followed by 40 cycles of 95°C for 10 seconds, and 60°C for 1 minutes. The comparative Ct method (ΔΔCt method) was used to analyze the relative expression values for each transcript using *Hprt* as a reference gene.

### In vivo 2-photon imaging and assessment of microglial process dynamics

Cx3CR1^+/GFP^ microglia reporter mice were anesthetized using fentanyl. As previously has reported [31], fentanyl did not influence microglial process motility compared to the effects of different anesthetics. Cranial window with 3 mm diameter was opened on the left hemisphere centered 1.5 mm lateral and 1 mm posterior to bregma without hurting the dura mater. After removal of the skull bone, a 3-mm and 5-mm double glass coverslip construct was fixed with 3M Vetbond tissue glue on top of the dura mater. Then, a custom-made metal headpiece (Femtonics, Budapest, Hungary) was fixed with dental cement on the surface of the skull. All experiments were performed on a Femto2D-DualScanhead microscope (Femtonics, Budapest, Hungary) coupled with a Chameleon Discovery laser (Coherent, Santa Clara, USA). Following excitation, the fluorescent signal was collected using a Nikon 18X water immersion objective. Data acquisition was performed by MES software (Femtonics). Galvano Z-stacks of 8 images (500 × 500 pixels, 3 μm step size, range = 100 to 125 μm from pial surface) were made at every minute. Two-photon image sequences were exported from MES and analyzed using FIJI. Microglial baseline process velocity was measured on time-series images acquired with 2P microscopy. Following motion correction, images from the same region of Cx3CR1^+/GFP^ mice were analyzed with the Manual Tracking plugin of FIJI. We applied a local maximum centring correction method with a search square of 5 pixels. Pixel size was 0.65 μm/px. Processes were included in the measurement when they were clearly traceable for at least 10 minutes. To compare how fast microglia cells are responding to injuries, cortical lesion was formed using laser beam. Focal lesion was induced with a 1,040-nm fix laser, and the laser power was 300 mW for 1,000 ms. The area of focal lesion was defined by a circle with 7.5 to 12.5 μm radius originating from the focal point of the laser beam. Baseline process motility was recorded before focal lesion, followed by a cell recruitment phase, and it was all repeated 15 minutes later after bumetanide administration (0.3 mg/kg body weight, in 5 μl final volume) into the cisterna magna in a different (undisturbed) part of the cerebral cortex in each animal.

Then, based on the idea of the procedure proposed by Davalos and colleagues [45], we created an automated image processing pipeline using CellProfiler [88] to determine the proportion of area over time covered by microglia cells on each image. We defined 2 concentric circles so that the whole area of the lesion is surrounded by the outer one, and the focal point of the ablation is covered by the inner one. The same dimensions were used among all images in the data set. We chose 25 μm for the inner diameter and 130 μm for the outer diameter on experimental basis. Only cells inside this region were taken into account, excluding the inner site as there were autoflourescent artifacts detected there. To differentiate between cells (high-intensity areas) and background (low-intensity areas), an adaptive threshold value was calculated from the per-image median intensities. The coverage was then measured as the proportion of pixels classified as cell and the total number of pixels in the area. Coverage values in the initial (baseline) phase of the experiment kept stable, no remarkable oscillation was recorded in any cases. Based on the observed data, we assumed that coverage reaches a minimum value ϱ_min_ as a result of the injury, then at time *t*_*s*_, microglia cells start to extend their processes into the lesion area uniformly from its perimeter with velocity *v*, and saturate at a final coverage ϱ_*max*_ rom this assumption, we created a simple mathematical model that predicts the coverage values over time inside the investigated area.


$$C(t)=(\varrho_{max}-\varrho_{min})\frac{r_{o}^{2}-{\left(r_{o}-v(t-t_{s})\right)}^{2}}{r_{o}^{2}-r_{i}^{2}}+\varrho_{min}$$

In this equation, *r*_*i*_ and *r*_*o*_ denote the inner and outer radii of the ring-shaped region, respectively. See S7 Fig for a visual explanation of the model. For each measured coverage data set, we fitted this model by choosing values for *v*, *q*_*max*_ and *t*_*s*_ that minimize the mean squared difference between the observed and predicted values. To find the best fitting values, a grid search-based optimizer algorithm was used. We found that the model is able to describe the observed behavior of cell flow from the external areas inside the lesion, and it also gives an estimate for the cell’s mean velocity, which is in good agreement with those from the manual tracking of cell processes.

### Middle cerebral artery occlusion (MCAo)

To assess the functional contribution of microglial NKCC1 to ischemic brain injury, mice were subjected to a 45-minute long MCAo using a silicone-coated filament, as described earlier by Dénes and colleagues [89]. Surgery was performed under isoflurane (1.5% in a 30% O_2_ and 70% N_2_O gas mixture) anesthesia and core body temperature was tightly controlled (37°C ± 0.5°C) during the whole procedure using a homeothermic heating blanket. Laser Doppler flowmetry was used to validate the occlusion of the MCA. In brief, after a midline incision made on the ventral surface of the neck, the right common carotid artery (CCA) was isolated, and a silicone-coated monofilament (210 to 230 μm tip diameter, Doccol, Sharon, USA) was introduced to the left external carotid artery (ECA) and advanced along the internal carotid artery (ICA) to occlude the MCA. Animals were kept in a postoperative chamber at 26 to 28°C until the functional assessment having free access to mashed food and water. Neurological outcome was assessed at 24-hour reperfusion using corner test and the 5-point Bederson’s sensory motor deficit scoring system [90–92]. Briefly, the following scores were given: a 0, no motor deficit; 1, flexion of torso and contralateral forelimb when mouse was lifted by the tail; 2, circling to the contralateral side when mouse is held by the tail on a flat surface, but normal posture at rest; 3, leaning to the contralateral side at rest; 4, no spontaneous motor activity; 5, early death due to stroke. Functional outcome has also been assessed by a complex neurological scoring system to obtain a more comprehensive readout [93]. Results are expressed as composite neurological score. Composite scores range from 0 (healthy mice) to 56 (worst performance) by adding up scores from 13 categories as follows: hair (0 to 2), ears (0 to 2), eyes (0 to 4), posture (0 to 4), spontaneous activity (0 to 4), and epileptic behavior (0 to 12); and focal deficits: body symmetry (0 to 4), gait (0 to 4), climbing on a surface held at 45° (0 to 4), circling behavior (0 to 4), front limb symmetry (0 to 4), compulsory circling (0 to 4), and whisker response to a light touch (0 to 4).

Infarct volume and brain edema were calculated after 24-hour survival on cresyl violet stained coronal brain sections using ImageJ as described previously [94]. In brief, lesion volume was determined at 8 neuroanatomically defined coronal levels (between +2 mm rostral and −4 mm caudal to bregma) by integrating measured areas of damage and correcting for edema size. The predetermined inclusion criteria for analysis were as follows: decline in Doppler signal of at least 70%, no cerebral hemorrhages, and survival to 24 hours. Cerebral hemorrhage was identified postmortem by the presence of excessive bleeding on the external surface of the brain, typically close to the filament location.

### Immunohistochemistry

#### Perfusion, tissue processing, and immunostaining for histology

Mice were anesthetized and transcardially perfused with 0.9% NaCl solution for 1 minute, followed by 4% PFA in 0.1 M phosphate buffer (PB) for 40 minutes, followed by 0.1 M PB for 10 minutes to wash the fixative out. Blocks containing the primary somatosensory cortex and dorsal hippocampi were dissected, and coronal sections were prepared on a vibratome (VT1200S, Leica, Germany) at 50 μm thickness for immunofluorescent histological and 100 μm thickness for the automated morphological analysis. Sections were washed in 0.1 M PB, incubated in 10% and 30% sucrose containing 0.1 M PB for 3 and 12 hours, respectively. Then the samples were placed into cryovials, snap frozen in liquid nitrogen, and stored at −80°C for further use. For the free-floating immunohistochemical detection of NKCC1, each vial contained sections from NKCC1^fl/fl^ (WT), NKCC1^fl/fl ΔCx3CR1^ (KO), and NKCC1^−/−^ mice, which sections were marked by different cuts, enabling that all experimental parameters were completely identical for all samples. The sections were washed in TBS, blocked with a Mouse-on-Mouse blocker solution (MOM blocker, #BMK-2202, Vectorlabs) for 1 hour, washed in TBS 2 × 5 minutes, and washed with MOM diluent 2 × 5 minutes. The diluted anti-NKCC1 primary antibodies (NKCC1 Rb: 1:4,000, #13884-1-AP, Proteintech, NKCC1 M: 1:2,000, diluted in MOM diluent; DSHB) were preincubated for 48 hours with brain slices from NKCC1^−/−^ mice in order to remove the fraction of immunoglobulins that could potentially cause non-specific binding. After discarding these slices, the guinea pig anti-Iba1 antibody (#234004, Synaptic Systems) was added to reach a 1:1,000 final dilution. This antibody mixture was applied on the samples for 48 hours at 4°C during gentle shaking. After intensive washes in TBS, sections were incubated with a mixture of secondary antibodies (donkey anti-mouse Alexa 647, 1:1,000, #715-605-150; donkey anti-rabbit Alexa 488, 1:1,000, #711-546-152; donkey anti-guinea pig Alexa 594, 1:1,000, #706-586-148; Jackson ImmunoResearch, diluted in TBS) for 24 hours. After subsequent washes in TBS and 0.1 M PB, DAPI staining was performed and the sections were mounted on glass slides and covered with Diamond Antifade (#P36961, ThermoFisher) or Aquamount (#18606–5, Polysciences). Immunofluorescence was analyzed using a Nikon Eclipse Ti-E inverted microscope (Nikon Instruments Europe B.V., Amsterdam, the Netherlands), with a CFI Plan Apochromat VC 60X oil immersion objective (numerical aperture: 1.4) and an A1R laser confocal system. We used 405, 488, 561, and 647 nm lasers (CVI Melles Griot), and scanning was done in line serial mode. Single-image planes were exported from the ND2 files, the outline of microglial cells was drawn using the Iba1-labeling, and the integrated density of the NKCC1 fluorescence signal was measured within these respective ROIs. This section is related to Fig 2.

#### Perfusion, processing, and immunostaining of ischemic and LPS-injected tissues

In terminal ketamine-xylazine anesthesia (100 mg/kg-10 mg/kg), mice were transcardially perfused with 0.9% NaCl solution, followed by 4% phosphate-buffered PFA. Brains of microglial NKCC1 KO mice subjected to 45-minute MCAo were postfixed and cryoprotected overnight (in 4% phosphate-buffered PFA-10% sucrose solution), then immersed into a cryoprotective solution (10% sucrose in PBS) at least 2 hours before 25 μm coronal sections were cut using a sledge microtome. Immunofluorescent staining was performed on free-floating coronal brain sections. Brain sections were blocked with 5% normal donkey serum followed by overnight incubation at 4°C using the following mixture of primary antibodies: goat anti-IL-1β/ILF2 (1:250; #AF-401-NA, R&D Systems), rat anti-CD45 (1:250; #MCA1388, AbD Serotec), rabbit anti-P2Y12R (1:500; #55043AS, AnaSpec). After the incubation with the primaries, sections were washed several times in TBS and were incubated with mixture of corresponding secondary antibodies at room temperature for 2 hours. The following secondaries were used: donkey anti-goat CF568 (1:1,000; #20106, Biotium), donkey anti-rabbit Alexa 647 (1:1,000; #711-605-152, Jackson ImmunoResearch), donkey anti-rat Alexa 488 (1:1,000; #712-546-153, Jackson ImmunoResearch). Slices were washed in TBS and were mounted to microscope slides using Fluoromount-G (#0100–01, SouthernBiotech). Representative images were captured with a 20X objective (Plan Apo VC, numerical aperture: 0.75, FOV = 645.12 μm) on a Nikon A1R confocal system. Quantitative analysis was performed on widefield images, captured with a 20X objective (Plan, numerical aperture: 0.4) on a Nikon Eclipse Ti-E inverted microscope (Nikon Instruments Europe B.V., Amsterdam, the Netherlands). IL-1α and IL-1β positive cells in the penumbral region, P2Y12R positive and CD45 positive cells in the whole cortex were counted on 3–3 serial coronal sections for a given brain area. This methodical description is related to Fig 6.

The following primaries and secondaries were used for GFAP and AQP4 immunolabeling: chicken anti-GFAP (1:1,000, #173006, Synaptic Systems) and guinea pig anti-AQP4 (1:500, #429004, Synaptic Systems), donkey anti-chicken A594 (1:500, #703-586-155, Jackson ImmunoResearch) and donkey anti-guinea pig A647 (1:500, #706-606-148, Jackson ImmunoResearch). For imaging, fluorescent slide scanner (Panoramic MIDI 3D HISTECH) with 20X Plan-Apochromat objective was used. Raw integrated densities were automatically measured on all images in selected ROIs from the striatum, and then their per-animal average was calculated and used for statistical analysis. This methodical description is related to S2 and S6 Figs.

### Electrophysiology

#### Hippocampal slices

Mice (56 to 65 days) were decapitated under deep isoflurane anesthesia. The brain was removed and placed into an ice-cold cutting solution The cutting solution contained (in mM): 205 sucrose, 2.5 KCl, 26 NaHCO_3_, 0.5 CaCl_2_, 5 MgCl_2_, 1.25 NaH_2_PO_4_, 10 glucose, saturated with 95% O_2_−5% CO_2_. Horizontal hippocampal slices of 250 μm thickness were cut using a Vibratome (Leica VT1000S). Slices were placed into an interface-type incubation chamber, which contained standard ACSF at 35°C that gradually cooled down to room temperature. At the beginning of the incubation period, a solution of 25 μM/ml Alexa 594 conjugated isolectin B4 (I21413, ThermoFisher) dissolved in ACSF was pipetted on top of each slice (15 ml/slice). After this, slice preparations were incubated in darkness for 1 hour before measurement. All measurements took place within 4 hours after slicing. The ACSF contained (in mM): 126 NaCl, 2.5 KCl, 26 NaHCO_3_, 2 CaCl_2_, 2 MgCl_2_, 1.25 NaH_2_PO4, 10 glucose, saturated with 95% O_2_−5% CO_2_.

#### Perforated patch recordings

After incubation for at least 1 hour, slices were transferred individually into a submerged-type recording chamber with a superfusion system allowing constantly bubbled (95% O_2_−5% CO_2_) ACSF to flow at a rate of 3 to 3.5 ml/min. The ACSF was adjusted to 300 to 305 mOsm (normotonic control solution), and the hypotonic solution was made by 50% dilution of control ACSF. Both normotonic and hypotonic solutions were constantly saturated with 95% O_2_−5% CO_2_ during measurements. All measurements were carried out at room temperature. A stock solution of 100 mg/ml gramicidin B (G5002, Sigma-Aldrich) diluted in DMSO was prepared daily and further diluted to 100 μg/ml concentration in the filtered pipette solution. We also added 100 μM Alexa Fluor 488 (A10436, ThermoFisher) to monitor membrane integrity during measurements. Before each recording, patch pipettes were fabricated from borosilicate glass and prefilled with gramicidin-free intracellular solution, and then backfilled with the intracellular solution containing gramicidin and Alexa 488. The pipette solution contained (in mM) the following: 120 KCl, 1 CaCl_2_, 2 MgCl_2_, 10 HEPES, and 11 EGTA (pH 7.3), 280 to 300 mOsm. Pipette resistances were 3 to 6 MΩ when filled with pipette solution. Visualization of slices and selection of cells (guided by isolectin B signal) was done under an upright microscope (BX61WI; Olympus, Tokyo, Japan, equipped with infrared-differential interference contrast optics and a UV lamp). Only cells located deeper than 15 to 20 μm measured from the slice surface were targeted. During recordings, Alexa 488 signal was constantly monitored to make sure that the membrane of cells did not suffer a rupture. Cells with an intracellular Alexa 488 signal were discarded. All cells were initially recorded in voltage-clamp mode at −40 mV holding potential. Series resistance was constantly monitored, and perforation was considered to be formed when the resistance fell to 35 to 50 MΩ (this regularly happened after 25 to 30 minutes after gigaseal formation). Individual recordings taken for analysis showed stability in series resistance and current response values between a 15% margin during the whole recording. After the perforated-patch configuration formed, resting membrane potential values were measured by changing the recording configuration to current-clamp mode at 0 pA for a short period of time (2 to 5 seconds). Thereafter, current responses to a pulse train of voltage steps from −140 mV to 60 mV with 20 mV increments were recorded in voltage-clamp mode with −40 mV holding potential. Each voltage step was 100 ms in duration and repeated 3 times. The interpulse interval was 2,000 ms. Pulse trains were recorded in the normotonic ACSF and after 5 minutes of perfusion with the hypotonic solution. Recordings were performed with a Multiclamp 700B amplifier (Molecular Devices). Data were digitized at 10 kHz with a DAQ board (National Instruments, USB-6353) and recorded with a custom software developed in C#.NET and VB.NET in the laboratory. For the extraction of current values, the last 40 ms of each voltage-step was used, during which the current responses were in a steady-state. Analysis was done using custom software developed in Delphi and Python environments. Input resistance of cells were determined by calculating the slope of I-V curves between the −40 mV and 0 mV voltage steps both in WT and KO.

### Automated morphological analysis of microglial cells

Mouse brains were cut into 100μm thick coronal slices and were immunostained with guinea pig anti-Iba1 (1:500; #234004, Synaptic Systems), Alexa 647 donkey anti-guinea pig (1:500; #706-606-148, Jackson ImmunoResearch) antibodies, and DAPI. Imaging was carried out in 0.1 M PB, using a Nikon Eclipse Ti-E inverted microscope (Nikon Instruments Europe B.V., Amsterdam, the Netherlands), with a CFI Plan Apochromat VC 60X water immersion objective (numerical aperture: 1.2) and an A1R laser confocal system. For 3D morphological analysis of microglial cells, the open-source MATLAB-based Microglia Morphology Quantification Tool was used (available at https://github.com/isdneuroimaging/mmqt). This method uses microglia and cell nuclei labeling to identify microglial cells. Briefly, 59 possible parameters describing microglial morphology are determined through the following automated steps: identification of microglia (nucleus, soma, branches) and background, creation of 3D skeletons, watershed segmentation, and segregation of individual cells [47].

### Quantification and statistical analysis

All quantitative assessment was performed in a blinded manner. Based on the type and distribution of data populations (examined with Shapiro–Wilk normality tests), we applied appropriate statistical tests: In the case of 2 independent groups, Student *t* test or Mann–Whitney U-test was applied; for 3 or more independent groups, one-way ANOVA followed by Tukey’s post hoc comparison or Kruskal–Wallis test with Dunn’s multiple comparison test was applied. Data were analyzed using the GraphPad Prism version 8.2 for Windows software (GraphPad Software, San Diego, California, USA). In this study, data are expressed as mean ± SEM, *p* < 0.05 was considered statistically significant.

## Supporting information

S1 Fig Effects of systemic vs. central blockade of NKCC1 on LPS-induced cytokine responses in the periphery. **(A)** ip. Bum injections further enhance the ip. LPS-induced G-CSF, IL-1α, and IL-1β production, while cor. LPS alone or with Bum has no significant effect on cytokine levels in the spleen and liver. (**B)** cor. LPS injection does not induce cytokine production, and Bum has no effect on baseline cytokine levels in the spleen and liver. (**C, D)** Flow cytometric dot plots show that cortical administration of Bum does not alter the numbers of CD4^+^ (P3 gate) and CD8^+^ (P4 gate) T cells and CD19^+^ MHCII^+^ (P6 gate) B cells in the spleen. All data are expressed as mean ± SEM. **(A)** One-way ANOVA followed by Sidak’s multiple comparison test (spleen) and Kruskall–Wallis test followed by Dunn’s multiple comparison test (liver); *p < 0.05; **p < 0.01; ***p < 0.001; *N* (veh.) = 5, *N* (veh. + ip. LPS) = 5, *N* (Bum + ip. LPS) = 5, *N* (veh. + cor. LPS) = 6, *N* (Bum + cor. LPS) = 9. (**B)** One-way ANOVA followed by Holm–Sidak’s multiple comparison test *N =* 6/group. (**D)** Kruskall–Wallis test followed by Dunn’s multiple comparison test; *N* (veh.) = 4, *N* (cor. LPS) = 4, *N* (cor. Bum + LPS) = 5. Data underlying this figure can be found in S1 Data. Bum, bumetanide; cor., cortical; ip., intraperitoneal; ns, not significant; veh., vehicle.
(TIF)

S2 Fig Intracortical blockade of NKCC1 does not alter astroglial GFAP or AQP4 levels. **(A, B)** CLSM images show immunolabeling for GFAP (yellow) and AQP4 (cyan) in NKCC1^fl/fl^ animals 24 hours after cortical injection of LPS or LPS + Bum and in the corresponding contralateral areas. (**C)** Raw integrated densities were automatically measured on all images in randomly selected ROIs from the injected ipsilateral cortical and contralateral regions prior to statistical analysis. No statistically significant difference in GFAP and AQP4 expression levels is seen in parenchymal astrocytes or perivascular astrocyte endfeet. (**B)** Scale: 25 μm. (**C)** One-way ANOVA followed by Holm–Sidak’s multiple comparisons test; *N =* 4 mice/group and 3–3 ROIs/animal. Data underlying this figure can be found in S1 Data. Bum, bumetanide; LPS, lipopolysaccharide; ns, not significant; ROI, region of interest.
(TIF)

S3 Fig Microglial NKCC1 deficiency does not alter cytokine levels and main leukocyte populations in the spleen after intracortical LPS injection. **(A, B)** Cytokine levels in the spleen and liver do not differ between WT and NKCC1 KO mice after intracortical LPS administration. **(C)** Numbers of CD4^+^ (P4 gate), CD8^+^ (P5 gate) T cells, and CD19^+^ MHCII^+^ B cells (P6 gate) are not altered in the spleen of NKCC1 KO mice compared to WT. Microglial NKCC1 deficiency does not affect the proportion of monocytes (P8 gate) or granulocytes (P7 gate) compared to WT. (**A, B)** Mann–Whitney test, *N* (WT) = 8, *N* (KO) = 6. (**C**) Unpaired *t* test; *N* (WT) = 4, *N* (KO) = 4. Data underlying this figure can be found in S1 Data. KO, knockout; ns: not significant; WT, wild type.
(TIF)

S4 Fig Deletion of NKCC1 from microglia results in a hyperpolarizing shift in the reversal of swelling-induced current. (**A)** I-V curves calculated by the subtraction of measured values in normotonic conditions from ones in hypotonic medium (WT: *N* = 8 cells, green with SEM; KO: *N* = 8 cells, blue with SEM), resulting in I-V curves representing the currents evoked by cell swelling due to osmotic change. (**B)** Reversal potentials of the swelling-induced currents measured from WT (green) or NKCC1 KO (blue) animals (left). Driving force was calculated for individual cells in WT (green) or KO (blue) by the subtraction of swelling-induced current reversal potentials from measured resting membrane potential (right). (**B)** Mann–Whitney; *N* (WT) = 8 cells, *N* (KO) = 8 cells; *: *p* < 0.05. Data underlying this figure can be found in S1 Data. KO, knockout; WT, wild type.
(TIF)

S5 Fig Changes in mRNA levels of microglial ion channels, transporters, and exchangers in the absence of microglial NKCC1 and after LPS treatment. **(A)** The expression of most genes that contribute to ion regulation, membrane potential, and cell volume regulation (anion channels (CLIC1); K^+^ channels (Kv1.3; Kir2.1; THIK-1; TWIK-2); ion exchangers (NH1E Na^+^/H^+^ exchanger; NCX1 Na^+^/Ca^2+^ exchanger; CLCN3 H^+^/Cl^−^ exchanger); and transporters (KCC3 K^+^/Cl^−^ transporter)) are not altered in NKCC1 KO microglia. However, Lrrc8d mRNA levels show a 2-fold increase in NKCC1 KO microglia cells. (**B**) *Slc9a1*, *Slc8a1*, *Lrrc8d*, *Clic1*, *Clcn3*, *Kcnk13*, *Kcnk6*, *Kcnj2*, *Sgk1* gene show decreased expression level in microglial cells 24 hours after intracisternal LPS injection. (**C**) *Slc9a1*, *Slc8a1*, *Slc12a6*, *Clic1*, *Clcn3*, *Kcnk13*, *Kcnk6*, *Kcnj2*, *Sgk1*, *P2RY12* gene did not show altered expression between WT and NKCC1 KO microglia after intracisternal LPS treatment. (**D**) Summary table of investigated genes. (**A-C**) Unpaired *t* test. (**A**) *N* (WT) = 6, *N* (KO) = 5; **: *p* < 0.01. (**B**) *N* (WT) = 6, *N* (WT + LPS) = 5, *: *p* < 0.05, **: *p* < 0.01, *** *p* < 0.001. (**C**) *N* (WT + LPS) = 5, *N* (KO + LPS) = 6, *: *p* < 0.05. Data underlying this figure can be found in S1 Data. KO, knockout; LPS, lipopolysaccharide; ns, not significant; WT, wild type.
(TIF)

S6 Fig Deletion of microglial NKCC1 does not alter astroglial GFAP and AQP4 levels. (**A, B)** CLSM images show immunolabeling for GFAP (yellow) and AQP4 (cyan) in microglial NKCC1 KO animals 24 hours after MCAo. (**C)** Raw integrated densities were automatically measured on all images in selected ROIs from the striatum, then, their per-animal average was calculated and used for statistical analysis. Data show no statistically significant differences in GFAP and AQP4 expression levels. (**B)** Scale: 25 μm. **(C)** One-way ANOVA followed by Holm–Sidak’s multiple comparisons tests; *N* (WT) = 7, *N* (KO) = 8 mice and 3–3 ROIs/animal. Data underlying this figure can be found in S1 Data. KO, knockout; MCAo, middle cerebral artery occlusion; ns, not significant; ROI, region of interest; WT, wild type.
(TIF)

S7 Fig Visual representation of the mathematical model of microglial process recruitment. (TIF)

S1 Video In vivo 2P time-lapse imaging of Cx3CR1^+/GFP^ mice shows microglial responses to focal lesion-induced injury under control conditions (left) and after cisterna magna bumetanide injection (right). Lesion-induced microglial process recruitment was determined by model fitting using image data from the circular region marked on the video. The outer perimeter of this region corresponds to the lesion site. Scale: 50 μm.
(AVI)

S1 Data Underlying numerical data for Figs 1A–1C, 1E, 2A, 2C, 2D, 2G, 3C–3E, 4A, 4D–4G, 5C–5G, 6B, 6C, and 6E and S1A, S1B, S1D, S2C, S3A–S3C, S4A, S4B, S5A–S5C, and S6C. (XLSX)

## Abbreviations

BBB: blood–brain barrier
CCA: common carotid artery
CCC: cation-chloride cotransporter
ECA: external carotid artery
FBS: fetal bovine serum
G-CSF: granulocyte colony stimulating factor
ICA: internal carotid artery
IL-1α: interleukin-1α
IL-1β: interleukin-1β
IL-6: interleukin-6
ip.: intraperitoneal
KC: keratinocyte chemoattractant
KCC: K^+^- Cl^-^ cotransporter
LPS: lipopolysaccharide
MCAo: middle cerebral artery occlusion
M-CSF: macrophage colony-stimulating factor
NKCC1: Na^+^-K^+^-2Cl^−^ cotransporter
NLRP3: NLR family pyrin domain containing 3
PB: phosphate buffer
PBS: phosphate-buffered saline
qRT-PCR: real-time quantitative PCR
TNF-α: tumor necrosis factor α
VRAC: volume-regulated anion channel
