# Supplementary figures and images for: The NKCC1 ion transporter modulates microglial phenotype and inflammatory response to brain injury in a cell-autonomous manner
Source: PLoS Biol. 2022 Jan 27;20(1):e3001526. doi: 10.1371/journal.pbio.3001526 (PMC8856735; doi:10.1371/journal.pbio.3001526)

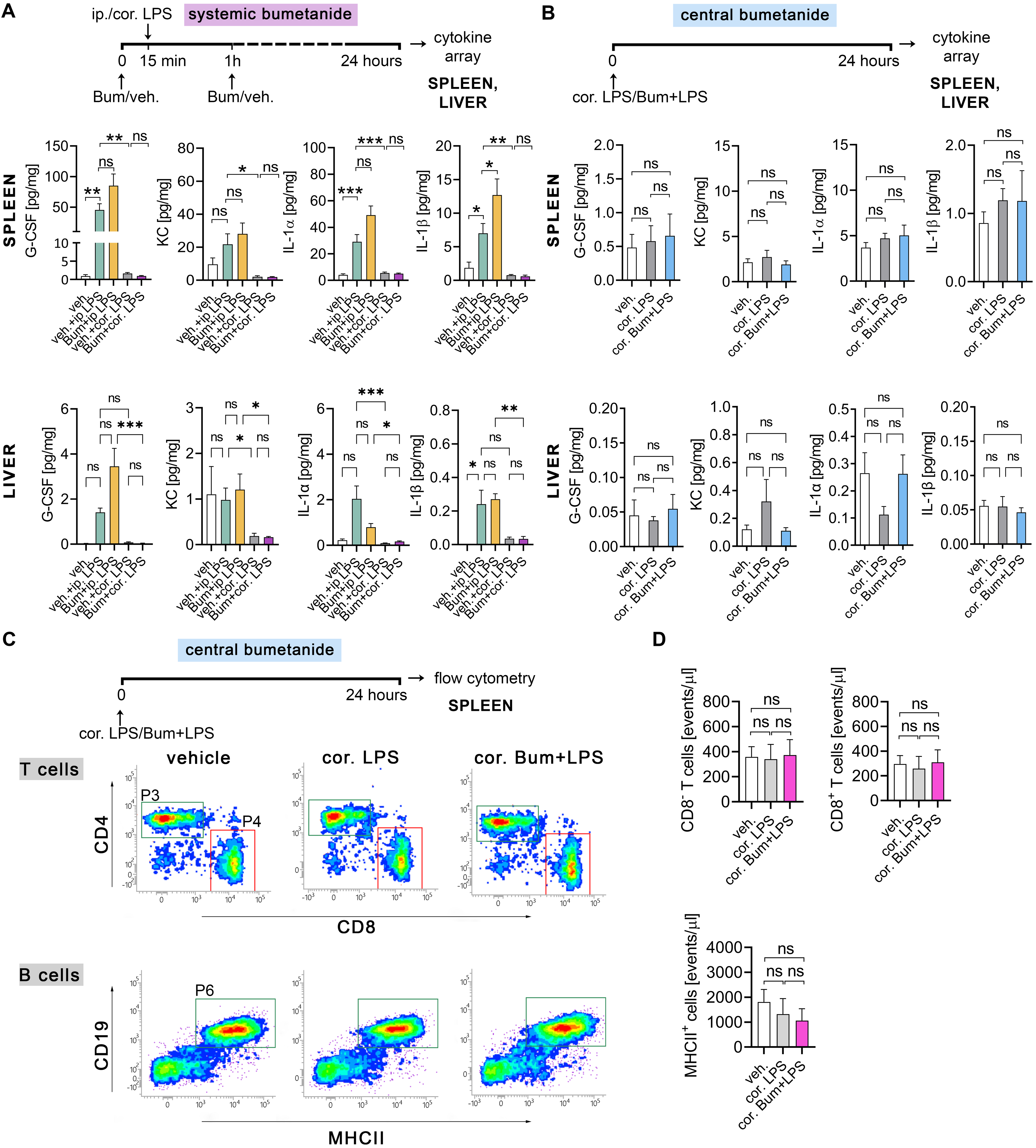

Supplement: S1 Fig — (A) ip. Bum injections further enhance the ip. LPS-induced G-CSF, IL-1α, and IL-1β production, while cor. LPS alone or with Bum has no significant effect on cytokine levels in the spleen and liver. (B) cor. LPS injection does not induce cytokine production, and Bum has no effect on baseline cytokine levels in the spleen and liver. (C, D) Flow cytometric dot plots show that cortical administration of Bum does not alter the numbers of CD4+ (P3 gate) and CD8+ (P4 gate) T cells and CD19+ MHCII+ (P6 gate) B cells in the spleen. All data are expressed as mean ± SEM. (A) One-way ANOVA followed by Sidak’s multiple comparison test (spleen) and Kruskall–Wallis test followed by Dunn’s multiple comparison test (liver); *p < 0.05; **p < 0.01; ***p < 0.001; N (veh.) = 5, N (veh. + ip. LPS) = 5, N (Bum + ip. LPS) = 5, N (veh. + cor. LPS) = 6, N (Bum + cor. LPS) = 9. (B) One-way ANOVA followed by Holm–Sidak’s multiple comparison test N = 6/group. (D) Kruskall–Wallis test followed by Dunn’s multiple comparison test; N (veh.) = 4, N (cor. LPS) = 4, N (cor. Bum + LPS) = 5. Data underlying this figure can be found in S1 Data. Bum, bumetanide; cor., cortical; ip., intraperitoneal; ns, not significant; veh., vehicle. (TIF) [file pbio.3001526.s001.tif]

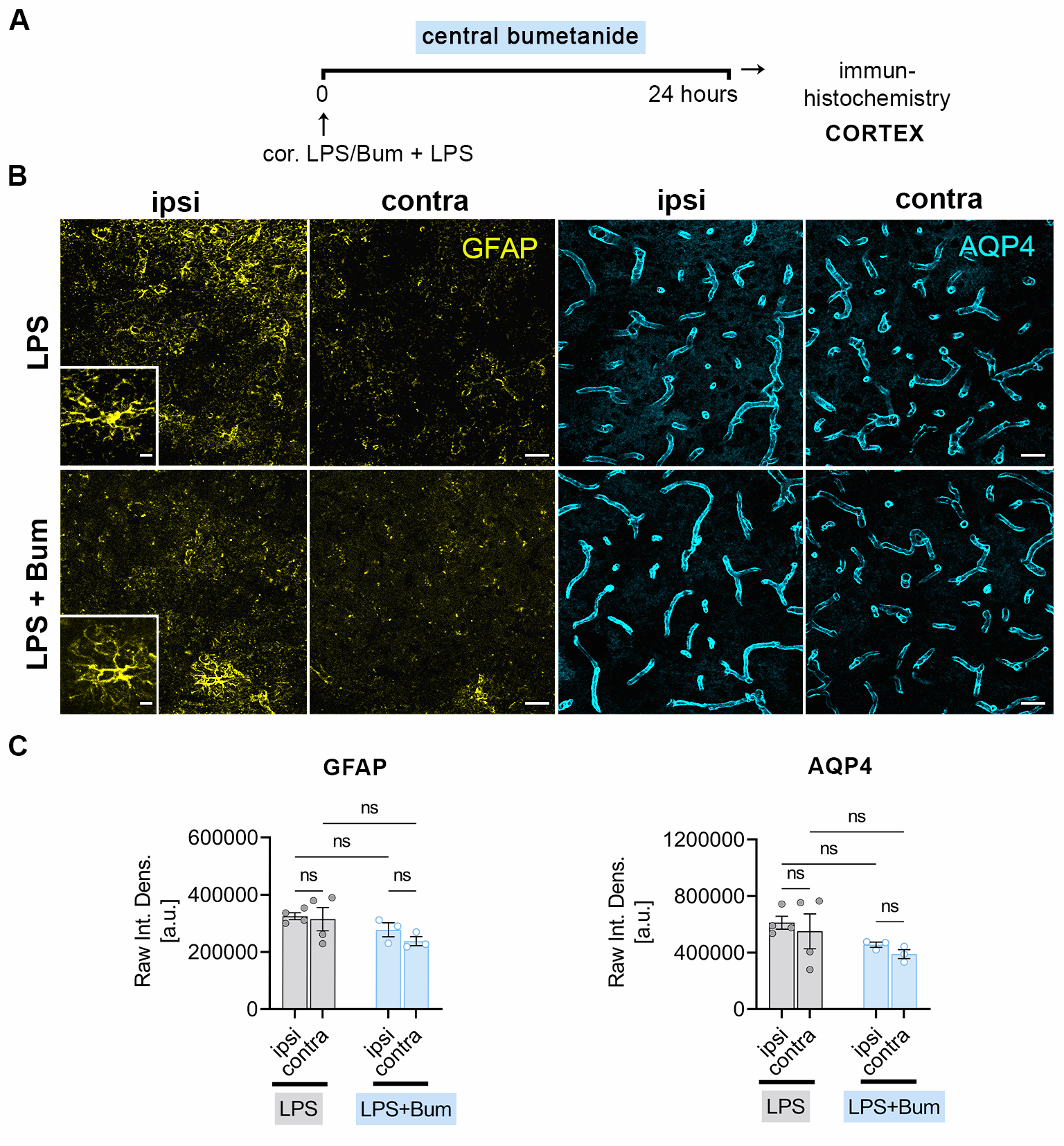

Supplement: S2 Fig — (A, B) CLSM images show immunolabeling for GFAP (yellow) and AQP4 (cyan) in NKCC1fl/fl animals 24 hours after cortical injection of LPS or LPS + Bum and in the corresponding contralateral areas. (C) Raw integrated densities were automatically measured on all images in randomly selected ROIs from the injected ipsilateral cortical and contralateral regions prior to statistical analysis. No statistically significant difference in GFAP and AQP4 expression levels is seen in parenchymal astrocytes or perivascular astrocyte endfeet. (B) Scale: 25 μm. (C) One-way ANOVA followed by Holm–Sidak’s multiple comparisons test; N = 4 mice/group and 3–3 ROIs/animal. Data underlying this figure can be found in S1 Data. Bum, bumetanide; LPS, lipopolysaccharide; ns, not significant; ROI, region of interest. (TIF) [file pbio.3001526.s002.tif]

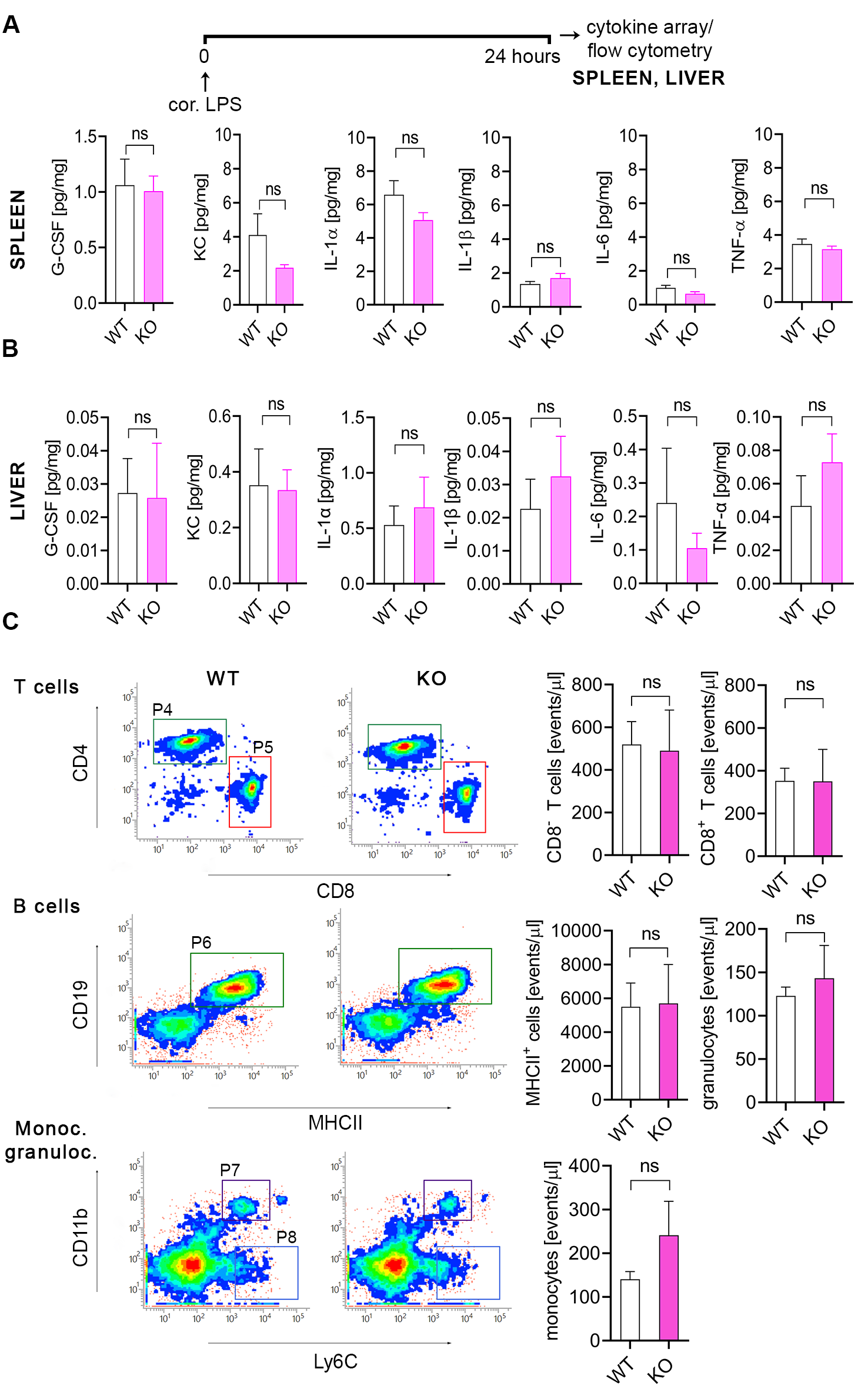

Supplement: S3 Fig — (A, B) Cytokine levels in the spleen and liver do not differ between WT and NKCC1 KO mice after intracortical LPS administration. (C) Numbers of CD4+ (P4 gate), CD8+ (P5 gate) T cells, and CD19+ MHCII+ B cells (P6 gate) are not altered in the spleen of NKCC1 KO mice compared to WT. Microglial NKCC1 deficiency does not affect the proportion of monocytes (P8 gate) or granulocytes (P7 gate) compared to WT. (A, B) Mann–Whitney test, N (WT) = 8, N (KO) = 6. (C) Unpaired t test; N (WT) = 4, N (KO) = 4. Data underlying this figure can be found in S1 Data. KO, knockout; ns: not significant; WT, wild type. (TIF) [file pbio.3001526.s003.tif]

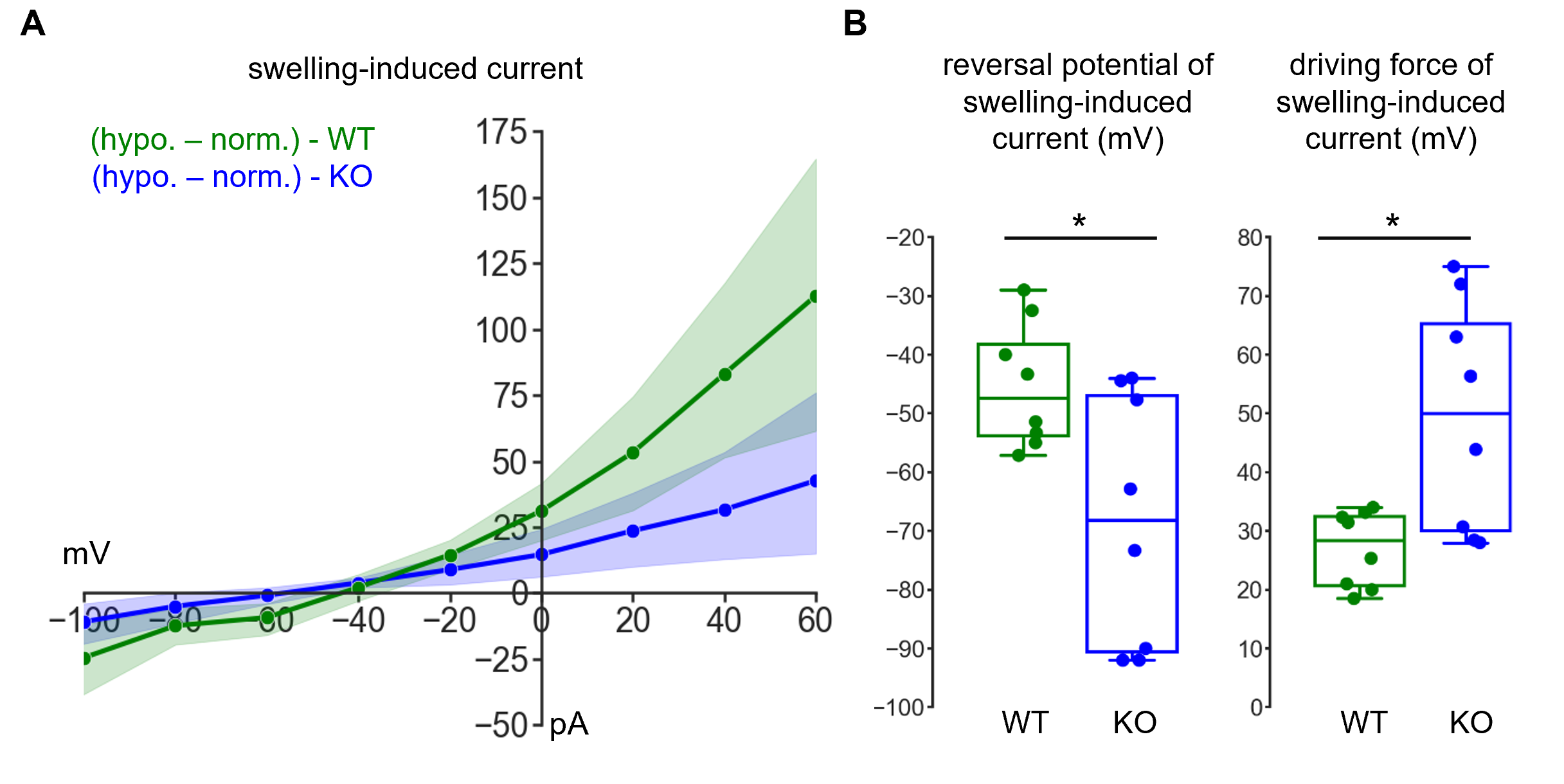

Supplement: S4 Fig — (A) I-V curves calculated by the subtraction of measured values in normotonic conditions from ones in hypotonic medium (WT: N = 8 cells, green with SEM; KO: N = 8 cells, blue with SEM), resulting in I-V curves representing the currents evoked by cell swelling due to osmotic change. (B) Reversal potentials of the swelling-induced currents measured from WT (green) or NKCC1 KO (blue) animals (left). Driving force was calculated for individual cells in WT (green) or KO (blue) by the subtraction of swelling-induced current reversal potentials from measured resting membrane potential (right). (B) Mann–Whitney; N (WT) = 8 cells, N (KO) = 8 cells; *: p < 0.05. Data underlying this figure can be found in S1 Data. KO, knockout; WT, wild type. (TIF) [file pbio.3001526.s004.tif]

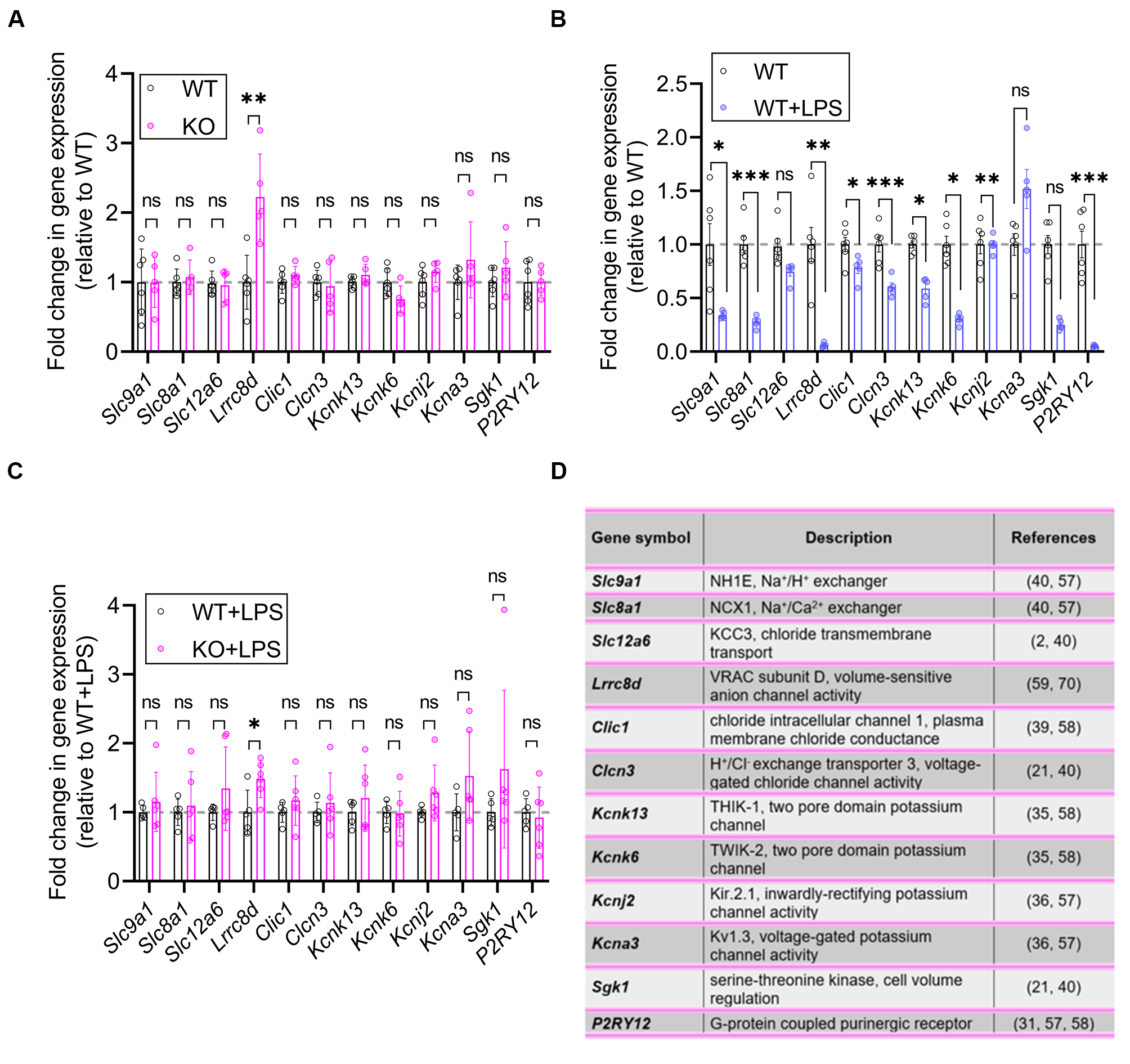

Supplement: S5 Fig — (A) The expression of most genes that contribute to ion regulation, membrane potential, and cell volume regulation (anion channels (CLIC1); K+ channels (Kv1.3; Kir2.1; THIK-1; TWIK-2); ion exchangers (NH1E Na+/H+ exchanger; NCX1 Na+/Ca2+ exchanger; CLCN3 H+/Cl− exchanger); and transporters (KCC3 K+/Cl− transporter)) are not altered in NKCC1 KO microglia. However, Lrrc8d mRNA levels show a 2-fold increase in NKCC1 KO microglia cells. (B) Slc9a1, Slc8a1, Lrrc8d, Clic1, Clcn3, Kcnk13, Kcnk6, Kcnj2, Sgk1 gene show decreased expression level in microglial cells 24 hours after intracisternal LPS injection. (C) Slc9a1, Slc8a1, Slc12a6, Clic1, Clcn3, Kcnk13, Kcnk6, Kcnj2, Sgk1, P2RY12 gene did not show altered expression between WT and NKCC1 KO microglia after intracisternal LPS treatment. (D) Summary table of investigated genes. (A-C) Unpaired t test. (A) N (WT) = 6, N (KO) = 5; **: p < 0.01. (B) N (WT) = 6, N (WT + LPS) = 5, *: p < 0.05, **: p < 0.01, *** p < 0.001. (C) N (WT + LPS) = 5, N (KO + LPS) = 6, *: p < 0.05. Data underlying this figure can be found in S1 Data. KO, knockout; LPS, lipopolysaccharide; ns, not significant; WT, wild type. (TIF) [file pbio.3001526.s005.tif]

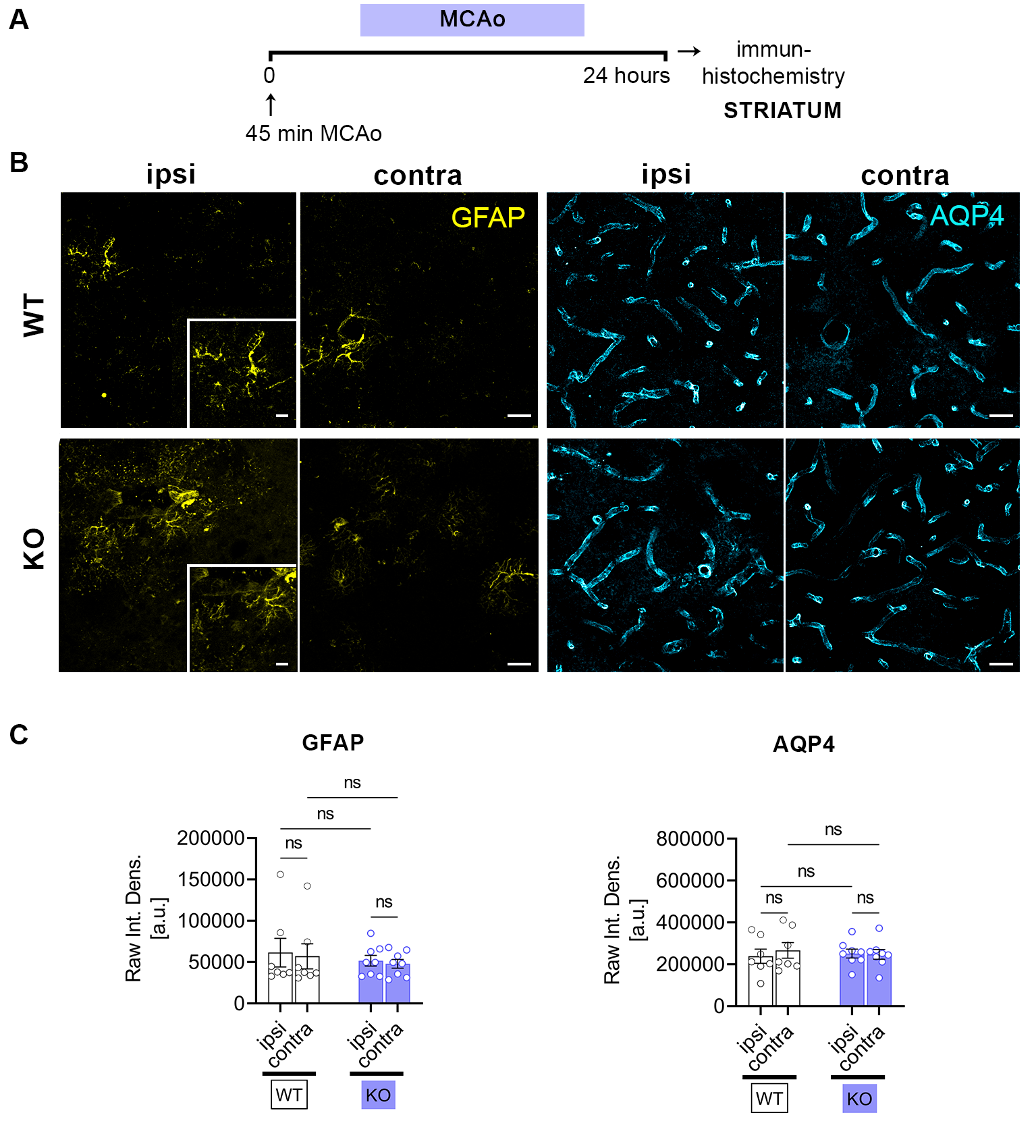

Supplement: S6 Fig — (A, B) CLSM images show immunolabeling for GFAP (yellow) and AQP4 (cyan) in microglial NKCC1 KO animals 24 hours after MCAo. (C) Raw integrated densities were automatically measured on all images in selected ROIs from the striatum, then, their per-animal average was calculated and used for statistical analysis. Data show no statistically significant differences in GFAP and AQP4 expression levels. (B) Scale: 25 μm. (C) One-way ANOVA followed by Holm–Sidak’s multiple comparisons tests; N (WT) = 7, N (KO) = 8 mice and 3–3 ROIs/animal. Data underlying this figure can be found in S1 Data. KO, knockout; MCAo, middle cerebral artery occlusion; ns, not significant; ROI, region of interest; WT, wild type. (TIF) [file pbio.3001526.s006.tif]

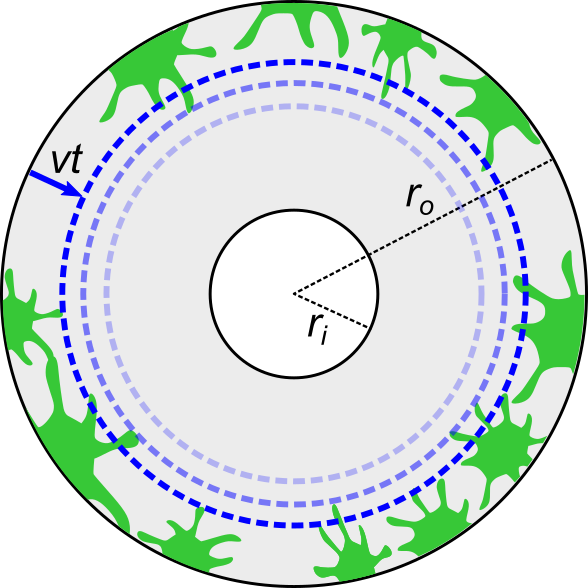

Supplement: S7 Fig — (TIF) [file pbio.3001526.s007.tif]
